# High‐Performance, Strain‐Stable Electromagnetic Shielding Materials Enabled by Magnetic Elastic Fiber Networks Pinning Liquid Metal

**DOI:** 10.1002/advs.202510078

**Published:** 2025-07-18

**Authors:** Qi Zhang, Yuanzhao Wu, Xilai Bao, Shengbin Li, Xueheng Zhuang, Zidong He, Jinyun Liu, Wuxu Zhang, Shiying Li, Feng Xu, Chuibin Zeng, Chao Hu, Qikui Man, Jie Shang, Yiwei Liu, Run‐Wei Li

**Affiliations:** ^1^ Ningbo Institute of Materials Technology and Engineering Chinese Academy of Sciences Ningbo 315201 P. R. China; ^2^ College of Materials Science and Opto‐Electronic Technology University of Chinese Academy of Sciences Beijing 100049 P. R. China; ^3^ Eastern Institute of Technology Ningbo 315200 P. R. China

**Keywords:** electromagnetic interference shielding, ferromagnetic nanofiber networks, liquid metal, magnetoelectric synergy effect, pinning‐interlocking mechanism

## Abstract

Stretchable electromagnetic interference (EMI) shielding materials are critical for the reliability of wearable electronic devices in complex electromagnetic environments. However, achieving compatibility between ultra‐thinness, high shielding efficiency (SE), and excellent dynamic stability remains a major challenge in this field. Here, an ultrathin elastic EMI shielding film (TPU/Fe‐LM) is developed by leveraging the magnetoelectric synergy effect and a pinning‐interlocking mechanism between ferromagnetic elastic nanofiber networks and the embedded liquid metal (LM), achieving high EMI SE and excellent strain stability. The ultrathin film, with a thickness of 85 µm, exhibits an average EMI SE exceeding 70 dB across a broad frequency range of 0.1 MHz to 40 GHz, with only a 2.59% variation under 100% tensile strain. This superb EMI SE per unit thickness (SSE = 1225 dB mm^−1^ @ 100% strain) ranks among the highest reported for stretchable EMI shielding films, highlighting the exceptional application potential. As a proof of concept, the EMI shielding film is integrated into a stretchable capacitive strain sensor for dynamic and static force sensing, achieving a 50‐fold enhancement in angle resolution for robotic motion monitoring. This research paves the way for stretchable EMI shielding materials and offers valuable guidance for enhancing electromagnetic protection in wearable electronics.

## Graphical abstract

An ultrathin elastic EMI shielding film (TPU/Fe‐LM) is developed by leveraging the magnetoelectric synergy effect and a pinning‐interlocking mechanism between ferromagnetic elastic nanofiber networks and embedded liquid metal, achieving high EMI SE and excellent strain stability. It exhibits an average EMI SE exceeding 70 dB from 0.1 MHz to 40 GHz, with only a 2.59% variation under 100% tensile strain.

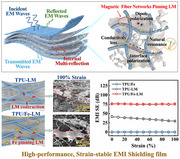

## 1 Introduction

High‐frequency and highly integrated electronic devices have sparked a groundbreaking surge in 5G communication technology. However, the resulting severe electromagnetic radiation and electromagnetic interference (EMI), which significantly poses a threat to reliability and normal operation of electronic devices,^[^ ^1^, ^2^, ^3^ ^]^ urgently demand effective solutions. Integrating high‐performance EMI shielding materials with electronic devices to address these challenges is considered an effective approach.^[^ ^4^, ^5^, ^6^ ^]^ With the advancement of wearable and human‐computer interaction technologies, there is a pressing demand for elastic EMI shielding materials with ultra‐thinness form (thickness < 100 µm) and stability of high EMI shielding effectiveness (SE) under tensile deformation, to achieve conformal integration on complex surfaces and adaptability to multidimensional dynamics.^[^ ^7^, ^8^, ^9^, ^10^, ^11^ ^]^

EMI shielding materials reduce interference by reflecting or absorbing electromagnetic waves (EMW), which results from the interaction between the electromagnetic field with mobile charge carriers and electric/magnetic dipoles in the shielding materials.^[^ ^12^ ^]^ Among these, the conductivity of the material is considered as a key factor to influence the shielding reflection. Currently, the research on stretchable EMI shielding materials primarily focuses on composite conductive materials, such as combining metal nanoparticles,^[^ ^13^, ^14^ ^]^ nanowires,^[^ ^15^ ^]^ carbon‐based materials (such as carbon black, graphene, and carbon nanotubes),^[^ ^16^, ^17^, ^18^ ^]^ 2D transition metal carbides (MXenes),^[^ ^19^, ^20^, ^21^ ^]^ and liquid metal (LM)^[^ ^22^, ^23^, ^24^, ^25^ ^]^ with elastic polymer matrices. Compared to other solid conductive fillers, gallium‐based LM stands out as a highly promising material for creating efficient stretchable EMI shielding materials due to their excellent deformability and high electrical conductivity (3.4 × 10^6^ S m^−1^). Moreover, previous studies have proved that LM composites exhibit excellent EMI shielding properties. For instance, Wang et al.^[^ ^24^ ^]^ demonstrated that a 3D LM/Ecoflex composite film with a thickness of 2 mm, across the frequency range of 8.2‐12.4 GHz, exhibited an EMI SE ranging from 34.5 to 86.2 dB when stretched up to 400%. Using LM as a filler in an elastomeric matrix is a simple and effective approach to fabricate stretchable EMI shielding materials. According to Simon's equation,^[^ ^26^ ^]^ constructing a conductive percolation network and increasing material thickness can improve EMI shielding performance. However, excessive LM content in forming a conductive percolation network is prone to leakage problems, while large thickness limits the application of LM based EMI shielding materials in wearable devices. Therefore, developing ultra‐thin and high‐performance stretchable EMI shielding materials with low LM content remains a challenge.

Enhancing the interaction between magnetic dipoles and the electromagnetic field by introducing magnetic particles is another effective strategy to improve EMI shielding performance.^[^ ^27^, ^28^, ^29^, ^30^ ^]^ Efforts have been made to enhance shielding performance by combining LM with magnetic particles. For example, Zhao et al.^[^ ^28^ ^]^ developed a LM magnetic hydrogel (with a thickness on the millimeter scale) that improved the initial EMI SE from 46.1 to 65.8 dB by utilizing the magnetoelectric synergistic effect of LM and nickel particles. Similarly, Zhang et al.^[^ ^30^ ^]^ developed a stretchable LM@Ni/Ecoflex composite with an SE of 60 dB at 75% strain and 35 dB at 300% strain. However, directly combining LM and magnetic particles reduces the deformability of the LM, compromising the stability of high shielding performance under tensile strain. Additionally, alloying reactions between the two materials can lead to transition in the crystalline structure of the magnetic materials,^[^ ^31^ ^]^ undermining the long‐term stability of EMI shielding performance. Therefore, developing ultrathin stretchable EMI shielding materials with high EMI SE and excellent strain stability, which meets the demands of wearable device applications, remains a formidable challenge.

Here we present an ultrathin stretchable EMI shielding film (TPU/Fe‐LM) with high EMI SE and excellent strain stability by leveraging the magnetoelectric synergy effect and a pinning‐interlocking mechanism between ferromagnetic elastic nanofiber networks and the embedded LM. A 3D ferromagnetic elastic nanofiber network facilitates uniform wetting and spreading of LM, enabling high electrical conductivity of the film even at low LM loading. Simultaneously, magnetic loss of ferromagnetic particles further enhances the EMI shielding performance. The composite film, with a thickness of only 85 µm, achieves an average shielding effectiveness exceeding 70 dB across a wide frequency range from 0.1 MHz to 40 GHz. Moreover, the pinning‐interlocking effect between ferromagnetic particles and LM effectively inhibits interfacial delamination under tensile deformation. Consequently, the composite maintains structural integrity and exhibits consistent EMI shielding performance, with only a 2.59% variation under 100% tensile strain across the broad frequency range of 0.1 MHz to 40 GHz. As a proof of concept, integrating the EMI shielding films into a stretchable capacitive strain sensor markedly improves its resolution under both static and dynamic conditions. In a representative application, such as robotic motion monitoring, the angle resolution was enhanced from 10° to 0.2°, corresponding to a 50‐fold improvement. Our work demonstrates a breakthrough approach for developing stretchable EMI shielding materials with surpassing performance and providing a solid foundation for improving the reliability of stretchable electronic devices in complex electromagnetic environments.

## 2 Results and Discussion

### 2.1 Design of the Stretchable TPU/Fe‐LM Composite Film

**Figure 1:**
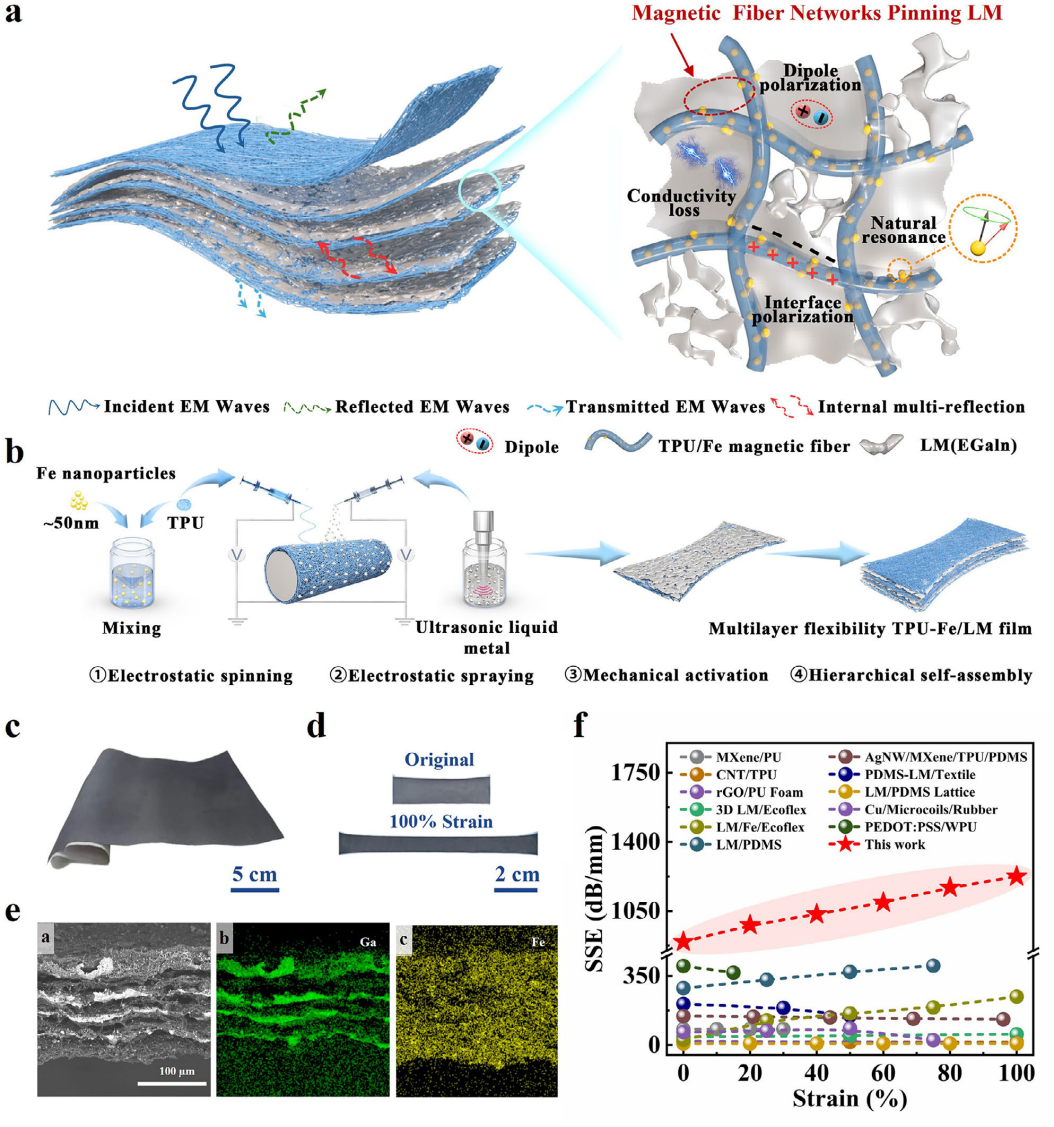
Design of stretchable TPU/Fe‐LM composite film with high EMI shielding performance. a) Schematic diagram of EMW loss in the stretchable EMI shielding composite film, resulting from the synergistic effects composed of conductivity loss, natural resonance, interface polarization, and multiple internal reflections. b) Preparation of stretchable TPU/Fe‐LM composite films by electrostatic spinning and spraying. c,d) The TPU/Fe‐LM composite film can be prepared over large areas and exhibits stretchable properties. e) Cross‐sectional SEM characterization and EDS element mappings of TPU/Fe‐LM composite film. f) Comparison of the SSE and strain stability of the TPU/Fe‐LM composite film with those of other stretchable EMI shielding films reported previously.

Based on the Schelkunoff formula,^[^ ^12^, ^32^ ^]^ EMI SE is positively correlated with both the electrical and magnetic permeability of the material. Hence, the magnetoelectric synergistic effect between highly conductive LM and high‐permeability ferromagnetic particles is anticipated to markedly enhance EMI SE. As shown in **Figure** **1**a, when an alternating electromagnetic field is applied to the TPU/Fe‐LM composite film, the enhanced EMI SE could be attributed to the following key mechanisms: 1) Conductivity loss: The highly conductive network formed by the LM generates significant induced currents, enabling the charge carriers to interact with EMW. This interaction inhibits EMW propagation by efficiently converting electromagnetic energy into thermal energy through ohmic loss. 2) Magnetic loss: The highly dispersed ferromagnetic particles in the fiber matrix form a low‐resistance magnetic path, effectively guiding and concentrating the magnetic field, which enhances EMW absorption through natural resonance. 3) Interfacial polarization: The heterogeneous interfaces between the LM and the TPU/Fe magnetic fibers lead to free charge accumulation at the interfaces, generating significant interfacial polarization loss. Additionally, multiple dipole active sites within the film enhance dipole polarization, further promoting the absorption of EMW. 4) Multiple internal reflections: The 3D porous network structure is composed of multiple magnetoelectric inhomogeneous interfaces between the LM and TPU/Fe magnetic fibers. This increases impedance mismatch, significantly enhancing the internal reflection of EMW.

The preparation process is detailed in Figure 1b. Stretchable TPU/Fe magnetic nanofibers (≈500 nm in diameter, Figure S1a, Supporting Information, shows the details) were fabricated via electrospinning. Subsequently, LM particles (ranging from nano to micrometer in size, see Figure S2, Supporting Information) were firmly adhered to the magnetic fiber skeleton using electrostatic spraying (Figure S1b, Supporting Information, shows the details). These components were assembled in multilayers to form a composite membrane with a robust 3D network structure. To establish the LM conductive pathways, the LM particles were activated in situ to form an ultrathin conductive layer, which was alternately distributed (as detailed in Figure S1c,d, Supporting Information). The X‐ray diffraction (XRD) spectra of the TPU/Fe‐LM composite films reveals prominent Fe diffraction peaks (Figure S3, Supporting Information), indicating that the magnetic iron nanoparticles are distributed within the fibers and exhibit high crystallinity. The Figure 1c,d demonstrate the large‐area and stretchability of the TPU/Fe‐LM composite film, which is revealed by the capability of being stretched to twice its initial length. As shown in the cross‐sectional scanning electron microscopy (SEM) images (Figure 1e), the film with a thickness of 85 µm possesses a multilayer structure consisting of a porous nanomagnetic fiber skeleton and LM multilayers. In a thorough comparison with previously reported stretchable EMI shielding materials,^[^ ^24^, ^25^, ^33^, ^34^, ^35^, ^36^, ^37^, ^38^, ^39^, ^40^, ^41^ ^]^ as depicted in Table S1 (Supporting Information), the TPU/Fe‐LM composite film exhibits significant advantages in terms of thickness, EMI SE and strain stability. Notably, the ultrathin TPU/Fe‐LM composite film stands out for its specific shielding effectiveness (SSE = SE/thickness), achieving an ultra‐high SSE of ≈1225 dB mm^−1^ under 100% tensile strain, as depicted in Figure 1f. This remarkable performance can be attributed to its ultra‐thinness and high SE.

### 2.2 EMI Shielding Properties of Composite Films

**Figure 2:**
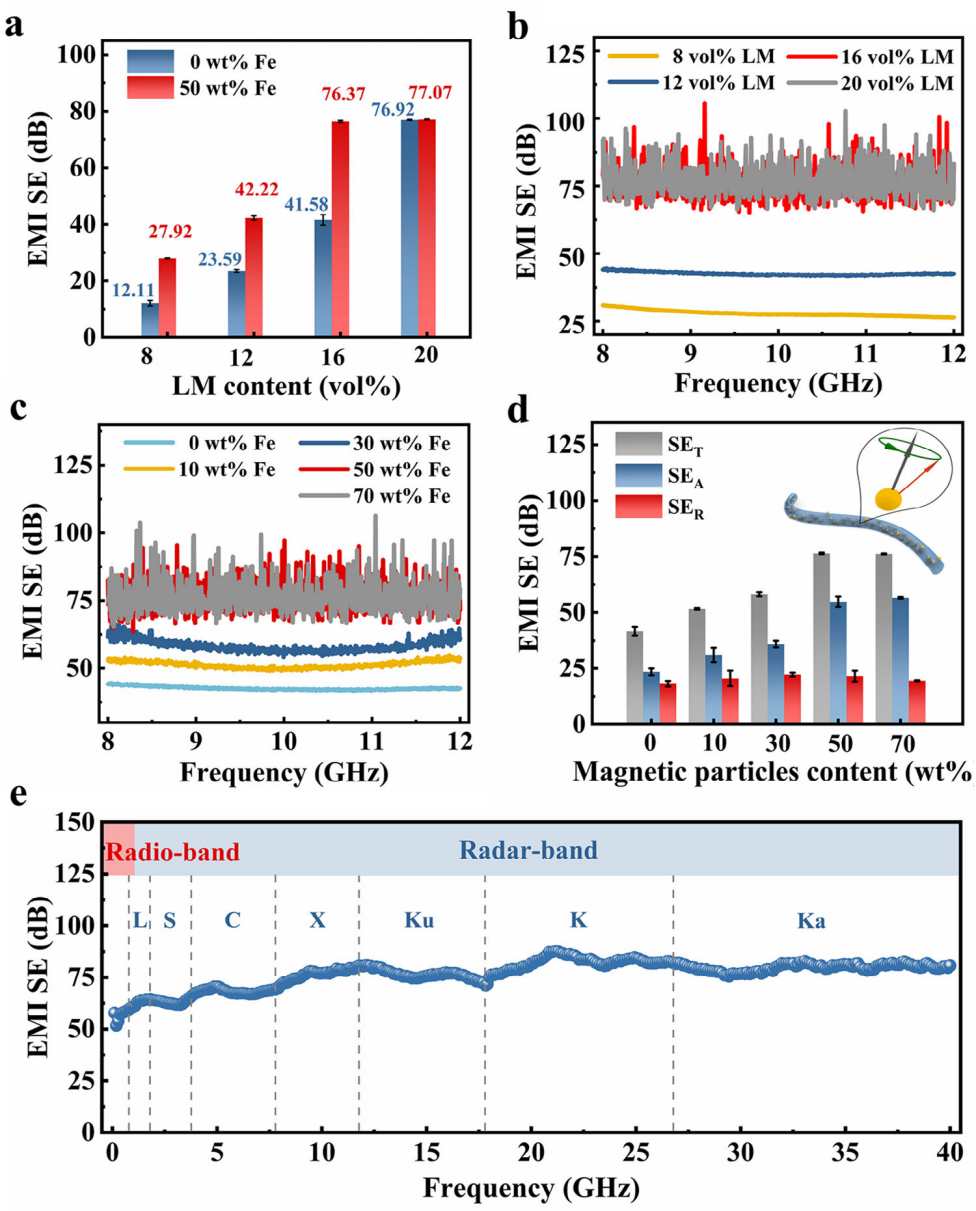
EMI shielding performance of TPU/Fe‐LM composite films with the thickness of 85 µm. a) Relationship between the LM content and the average EMI SE_T_ values of the films fabricated with/without 50 wt.% ferromagnetic particles, the data were averaged over a frequency range of 8.2–12.4 GHz. b) Total EMI SE curves of TPU/Fe‐LM composite films with different LM contents over the frequency range of 8.2–12.4 GHz (the ferromagnetic particle content of the films is fixed at 50 wt.%). c) Total EMI SE curves of films with different ferromagnetic particle contents over the frequency range of 8.2‐12.4 GHz (the LM content of the films is fixed at 16 vol%, the same in panels (d) and (e)). d) Average EMI SE_T_, SE_R_, and SE_A_ values of the films with different ferromagnetic particle contents, the data were averaged over a frequency range of 8.2–12.4 GHz. e) Total EMI SE curve of the TPU/Fe‐LM composite film over the frequency range of 0.1 MHz‐40 GHz (the ferromagnetic particle content of the films is fixed at 50 wt.%).

Electrical conductivity and magnetic permeability are critical in determining the EMI SE of the composite film, these properties significantly influence the reflection and absorption of EMW. To optimize EMI shielding performance via magnetoelectric synergy, we evaluated the EMI shielding performance of the prepared films by incorporating the same mass of ferromagnetic particles into all films with different LM contents. The EMI shielding performance of the film was assessed using the waveguide method. As shown in **Figure** **2**a and S4 (Supporting Information) the EMI shielding performance of the films is significantly enhanced by adding 50 wt.% ferromagnetic particles. For example, the EMI SE of the TPU‐LM film containing 8 vol% LM is only 12.11 dB, which increases to 27.92 dB after the incorporation of ferromagnetic particles, representing a 1.3‐fold improvement. The conductivity of the composite films increases significantly from 1.9 × 10^3^ to 4.7 × 10⁴ S m^−1^ when the LM volume fraction is increased from 8 vol% to 20 vol% (Figure S5, Supporting Information). This enhancement in conductivity is attributed to the increase in the number of mobile carriers within the films, facilitating the formation of continuous conductive pathways. The average total SE (SE_T_) of the TPU/Fe‐LM composite film increases from 27.92 dB at 8 vol% LM to 76.37 dB at 16 vol% LM, thereafter reaching a saturation. This improved shielding performance is consistently maintained across the full X‐band (with a frequency range of 8.2–12.4 GHz), as shown in Figure 2b. The SE_T_ of the composite film increases with increasing Fe content (with a constant LM volume percentage of 16 vol%), as shown in Figure 2c,d. When the Fe particle content increases from 0 wt.% to 70 wt.%, the SE_T_ increases from 41.58 dB to 76.13 dB. Simultaneously, the percentage of EMI SE absorption (SE_A_/SE_T_ ratio) increases from 56.38% to 74.31%, as shown in Figure S6 (Supporting Information).

To further analyze the EMI shielding mechanism of TPU/Fe‐LM films, the average values of reflectivity (*R*), absorptivity (*A*), and transmissivity (*T*) coefficients of films at X‐band frequency were analyzed. According to the law of power balance, *R *+ *A* + *T*  =  1.^[^ ^42^ ^]^ The results show that the *R* coefficient value decreases regularly with the increase of the mass percentage of ferromagnetic particles: the *R*‐value of the pure TPU‐LM film is 0.995, while the *R*‐value of the TPU/Fe‐LM film with 70 wt.% Fe content decreases to 0.982 (Figure S7, Supporting Information). It is noteworthy that although the reflection SE (SE_R_) is relatively low, *R* remains high due to the logarithmic relationship between SE_R_ and *R*.^[^ ^42^, ^43^ ^]^ Therefore, in this study, despite the *R*‐value was consistently higher than the *A*‐value, the absorption SE (SE_A_) was greater than SE_R_ for all samples.

Since the *T*‐value of TPU/Fe‐LM films is extremely close to 0, the dissipation of most of the EMW entering the internal structure of the film occurs primarily through the following effects: On the one hand, the LM, which wetting and spreading across the surface of the ferromagnetic nanofibers, forms a continuous conductive network that effectively converts electromagnetic energy into heat through ohmic losses.^[^ ^19^, ^43^ ^]^ Additionally, when the film is exposed to an alternating electromagnetic field, the free charge accumulates in large amounts at the interface between the LM and the TPU/Fe magnetic fibers, triggering a significant interfacial polarization loss.^[^ ^21^, ^25^ ^]^ Meanwhile, multiple magnetoelectric heterogeneous interfaces exist between the LM and the TPU/Fe magnetic fibers, which further increase the impedance mismatch and significantly enhance the multiple reflections and scattering of EMW inside the film.^[^ ^20^, ^22^ ^]^ Additionally, resonance occurs when the frequency of the external alternating magnetic field aligns with the precession frequency of the magnetic moments. At this point, the energy from the external magnetic field is transmitted to the material via ferromagnetic resonance. This process results in magnetic losses and significant absorption of magnetic energy, thereby attenuating the EMW.^[^ ^25^, ^28^, ^44^, ^45^ ^]^ Consequently, as the ferromagnetic particles content increases, the *R* coefficient values of the film decrease, but the loss of absorption capacity increases.

To investigate the magnetic loss mechanism of the TPU/Fe‐LM composite films, the hysteresis loops of films with different ferromagnetic particle contents were measured at room temperature, as shown in Figure S8 (Supporting Information). The results reveal that the saturation magnetization of the films is positively correlated with the Fe content, with values of 7.10 (Fe‐10 wt.%), 18.51 (Fe‐30 wt.%), 27.59 (Fe‐50 wt.%), and 43.06 (Fe‐70 wt.%) emu g^−1^, respectively. The low coercivity of the ferromagnetic particles indicates that they are easily magnetized, enabling the films to achieve significant magnetization under a small external magnetic field and respond rapidly to alternating electromagnetic fields. This property enhances the impedance mismatch and reduces EMW propagation through magnetic losses. Additionally, a higher ferromagnetic particle content amplifies the natural resonance effect between the composite film and EMWs, improving the efficiency of electromagnetic energy absorption. Furthermore, to demonstrate the magnetic loss mechanism, by adding the same mass (50 wt.%) of silver particles with the same particle size (≈50 nm) into the 8 vol% LM film, the conductivity of the TPU/Ag‐LM film is slightly higher than that of the TPU/Fe‐LM film, with values of 2.2 × 10^3^ and 1.9 × 10^3^ S m^−1^, respectively (Figure S9, Supporting Information). However, the EMI SE of the TPU/Fe‐LM film is 27.92 dB, significantly higher than the 14.59 dB of the TPU/Ag‐LM film. This confirms that the ferromagnetic particles enhance shielding performance via the natural resonance (Figure S10, Supporting Information). It is worth noting that although the introduction of ferromagnetic particles can improve magnetic loss, as the magnetic particle content increases, the film's elongation at break gradually decreases, as shown in Figure S11 (Supporting Information). Since ferromagnetic rigid particles are dispersed in the TPU matrix at a high‐volume fraction, the rigid phase limits the migration of the elastic molecular segments. The resulting uneven dispersion weakens interfacial bonding, leads to stress concentration, and significantly reduces the mechanical properties of the film.^[^ ^46^, ^47^ ^]^ To achieve an optimal balance between high shielding performance and tensile deformation, we selected 50 wt.% ferromagnetic particle content as the optimal composition, which results in a film elongation at break of 150% (unless otherwise specified later, the ferromagnetic particle content will be defaulted to 50 wt.%).

The film thickness influences the propagation path of EMW, which subsequently affects EMI shielding performance.^[^ ^20^, ^22^ ^]^ To investigate this effect, the variation in EMI shielding performance across different film thicknesses was studied. With the LM (16 vol%) and ferromagnetic particles (50 wt.%) content fixed, it was observed that as the film thickness decreases from 85 µm to 47 µm, the shielding effectiveness (SE) in the X‐band decreases from 76.37 dB to 56.77 dB, as shown in Figure S12 (Supporting Information). This behavior follows the approximate attenuation formula predicted by transmission line theory: $SE\propto t\sqrt{\pi f\mu \sigma }$,^[^ ^12^ ^]^ where *t*, *µ*, *σ*, and *f* represent the thickness, permeability, conductivity of the shielding material, and the frequency of incident EMW, respectively. Considering the conformal integration with wearable devices and the high EMI performance, 85 µm was selected as the optimal thickness. The composite film maintains a high average EMI SE of over 70 dB across the frequency range of 0.1 MHz to 40 GHz (Figure 2e), demonstrating high‐performance EMI SE over a broad frequency range. This broad coverage in frequency significantly expands its potential applications in ameliorating various electromagnetic signals.

**Figure 3:**
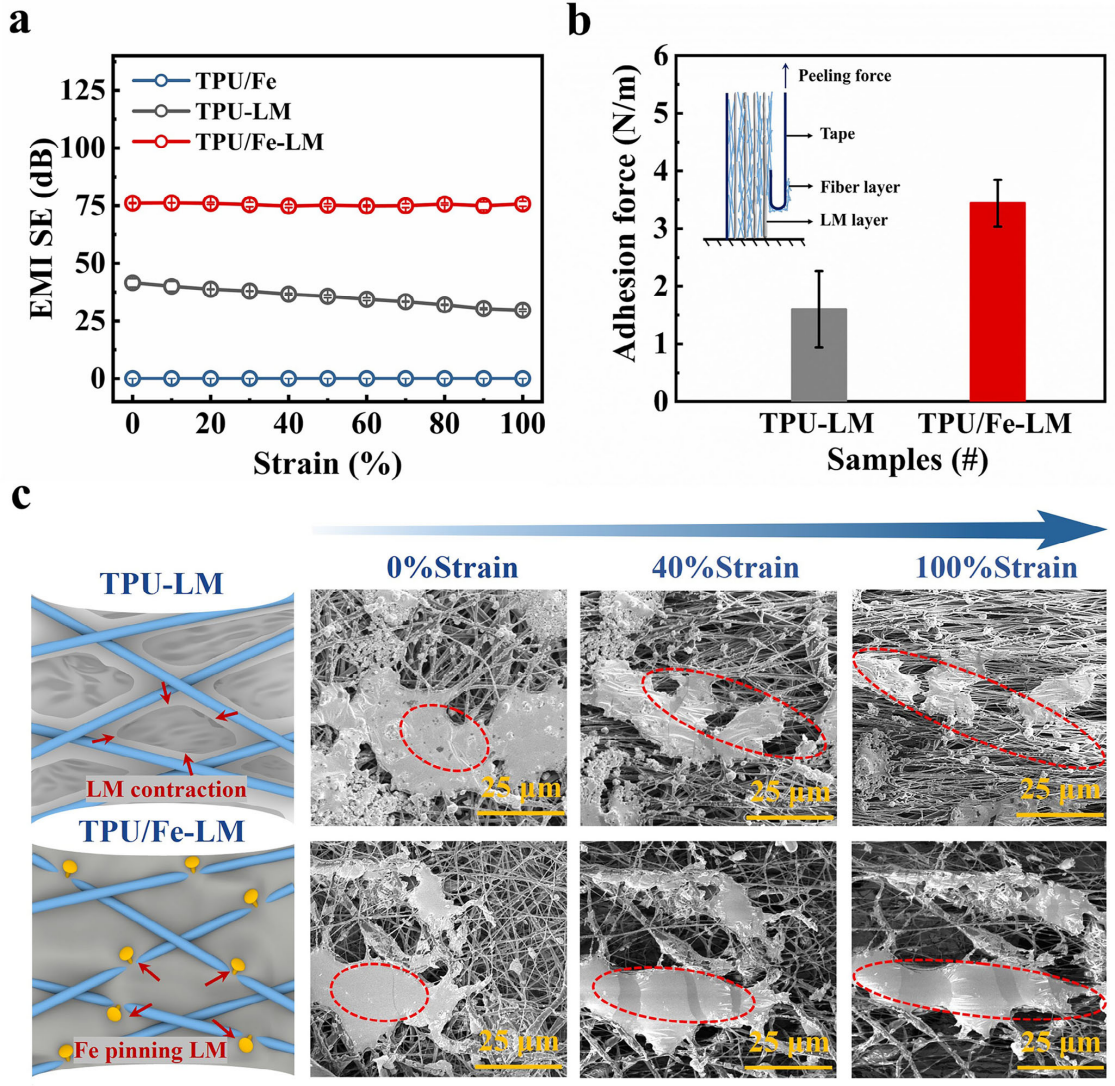
High‐performance, strain‐stable EMI shielding and pinning effect characterization. a) Average EMI SE_T_ values of TPU/Fe, TPU‐LM, and TPU/Fe‐LM composite films under different tensile strains. b) Adhesion force of TPU‐LM film and TPU/Fe‐LM film. c) In situ micromorphology analysis of TPU‐LM film and TPU/Fe‐LM film under 0%, 40%, and 100% strain stretching, the data were captured through SEM.

To meet the EMI shielding requirements for flexible electronics, the performance of the composite films under various strains was further investigated using a custom‐designed stretcher (Figure S13, Supporting Information). Observations in Figure S14 (Supporting Information) reveal that varying the stretching speed exerts negligible influence on the stress‐strain curves of the films. As shown in **Figure** **3**a, the EMI SE of the TPU/LM composite film (the film without magnetic particles) significantly decreases under tensile strain, from 41.58 dB in the initial state to 29.68 dB at 100% tensile strain under 8–12 GHz. Notably, the shielding performance of the composite film under tensile deformation is stabilized by the introduction of a moderate amount of ferromagnetic particles (content >50 wt.%), as shown in Figure 3a and Figure S15 (Supporting Information). A coaxial test system was utilized to fully test the SE of the films at 100% tensile strain over the wide frequency range of 0.1 MHz to 40 GHz. The results show that the TPU/Fe‐LM films exhibit excellent strain stability at 100% tensile strain. Initially, the average SE of the film is 76.21 dB over the entire test band, and when the tensile strain reaches 100%, the average SE decreases to 74.23 dB, a change of only 2.59%, which proves that the film has strain stability over a wide frequency range (Figure S16, Supporting Information). This excellent strain stability is attributed to the wetting and spreading of LM facilitated by the ferromagnetic nanofiber network and the pinning‐interlocking effect. The addition of ferromagnetic particles increased the surface roughness of the fibers from 1000 to 1274 nm, as shown in Figure S17 (Supporting Information). According to Wenzel's wetting model,^[^ ^48^, ^49^ ^]^ a rough surface significantly enhances the actual contact area between the LM and the magnetic elastic fiber, thereby increasing the interfacial free energy. The resulting driving force promotes the spreading of the LM along the fiber surface, ultimately forming a more stable 3D conductive network (Figures S18 and S19, Supporting Information). Moreover, the proliferation of nanostructures on the rough surface strengthens the mechanical interlocking between the LM and the fiber, forming “anchor‐like” pinning points. This interlocking effect significantly reduces interfacial slip between the LM and the substrate during deformation. The adhesion of the TPU/Fe magnetic fibers and TPU fibers to the LM was characterized using a 180° peeling test. The results show that the adhesion force between TPU/Fe magnetic fibers and the LM is approximately 3.4 N m^−1^, significantly higher than that between TPU fibers and the LM (1.59 N m^−1^), as shown in Figure 3b. This further demonstrates that the pinning‐interlocking effect of the ferromagnetic nanofiber network effectively enhances the adhesion strength with LM.

Meanwhile, we further investigated the surface morphology of films under tensile deformation by in situ SEM to evaluate the stability of LM based conductive network under strain, which is shown in Figures 3c, Figure S20 (Supporting Information). From these figures it can be depicted that the pores are prone to be generated in the TPU/LM film under tensile deformation due to the high surface tension of LM, which ultimately leads to the fracture of the LM film at larger tensile strains. This issue destabilizes the EMI SE in LM elastomer composites under deformation. In contrast, the TPU/Fe‐LM film, with ferromagnetic particles “pinning” the LM to the magnetic fibers, exhibits a more stable interface during deformation. This pinning effect reduces the risk of separation and generation of pores between LM and magnetic fibers under tensile deformation, thus keeping the conductive network intact at larger strains. Figure S21 (Supporting Information) shows that the relative electrical resistance change of the TPU/Fe‐LM film under tensile deformation is only 7%, compared to 106% for the TPU‐LM film. Electrical stability is key to maintaining the stability of EMI shielding performance under tensile deformation. Therefore, due to the pinning effect of ferromagnetic particles on LM in TPU/Fe‐LM films, the EMI shielding performance remains stable under tensile deformation.

**Figure 4:**
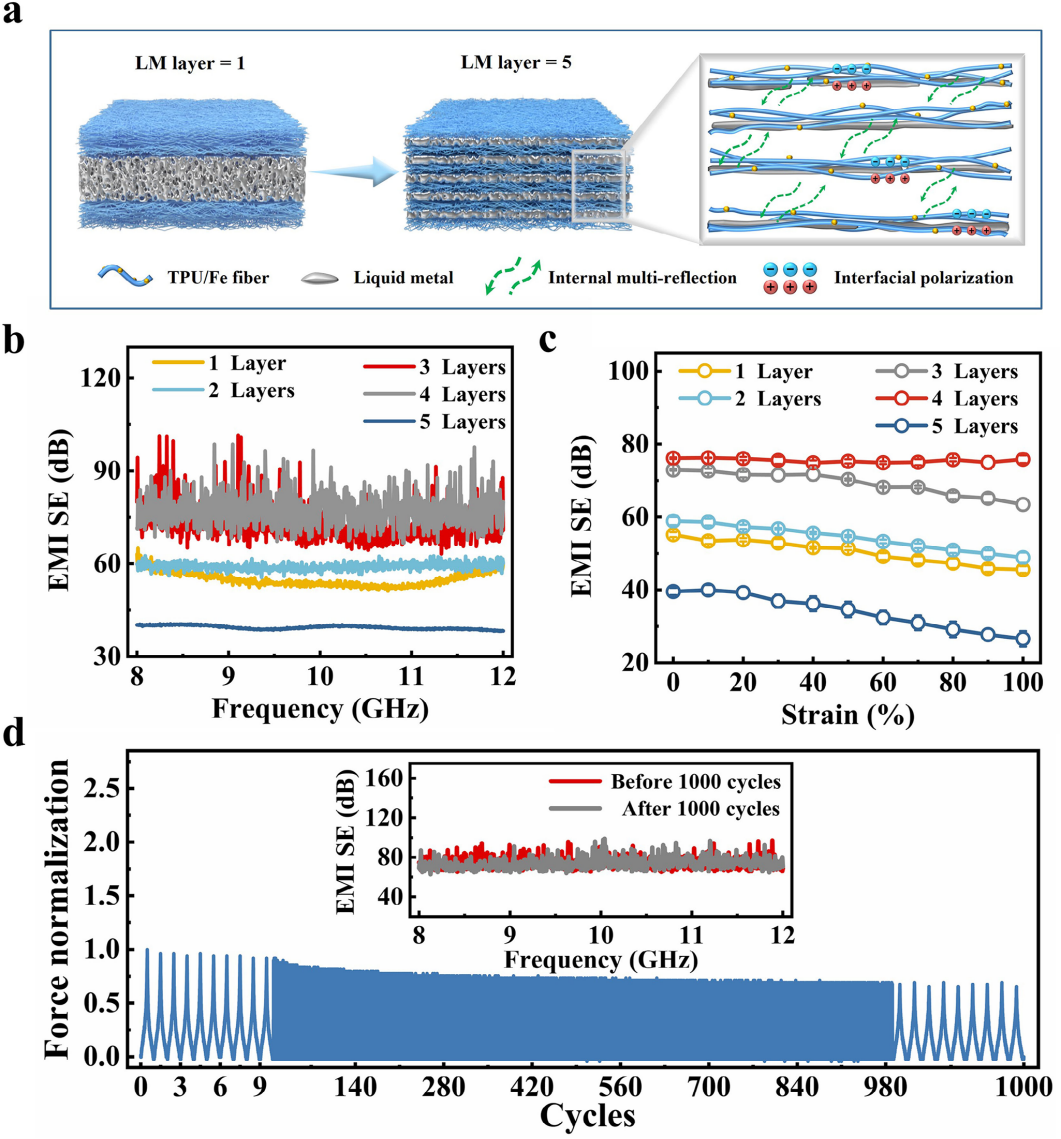
EMI shielding performance of TPU/Fe‐LM composite films with multilayered structures. a) Schematic diagram of multilayer film and its mechanism. b) EMI SE of TPU/Fe‐LM composite films with different number of LM layers. c) Average EMI SE_T_ values of the TPU/Fe‐LM composite films with different LM layers under different tensile strains. d) EMI shielding performance of TPU/Fe‐LM composite film under 1000 cycles of stretching and releasing under 50% tensile strain (4 layers LM).

Furthermore, the effect of different magnetoelectric heterogeneous interfaces on EMI shielding performance was investigated. This was accomplished by varying the number of LM layers while keeping the LM content, ferromagnetic particle content, and total film thickness constant (16 vol% LM, 50 wt.% Fe, 85 µm). A schematic illustrating the structure with different numbers of LM layers is shown in **Figure** **4**a. The results are presented in Figure 4b,c, where the initial EMI SE of the single‐layer LM composite film is 55.15 dB. As the number of LM layers increases, the number of heterogeneous interfaces between the LM and ferromagnetic particles increases, thus improving initial EMI SE through enhanced pinning‐interlocking effect and stronger magnetoelectric synergistic interactions. This also contributes to the improved stability of the EMI shielding performance under tensile deformation. However, in the 5 layers of the LM structure, the total amount of LM is evenly distributed across the layers. As a result, the LM content in each layer is substantially decreased. This reduction prevents the LM from reaching its percolation threshold, resulting in poor conductivity and thus leading to lower EMI performance (only 39.56 dB). Experimental results indicate that 4 layers of LM structure achieve a more favorable balance between the LM‐based conductive network and the number of heterogeneous interfaces, thus enhancing the EMI shielding performance.

In addition, maintaining robust electrical stability during stretching is crucial for enduring EMI shielding performance. Fatigue analysis further reveals that the EMI shielding performance of the material remains stable even after 1000 cycles at 50% strain (Figure 4d). This stability is attributed to the multilayer LM arrangement forms a stable 3D network through staggered and interwoven connections, effectively dispersing stress and enhancing the strain stability of the film. The shielding properties remain stable for up to one year, demonstrating exceptional long‐term stability (Figure S22, Supporting Information).

### 2.3 Application of Composite Film in Flexible Capacitance Sensors

**Figure 5:**
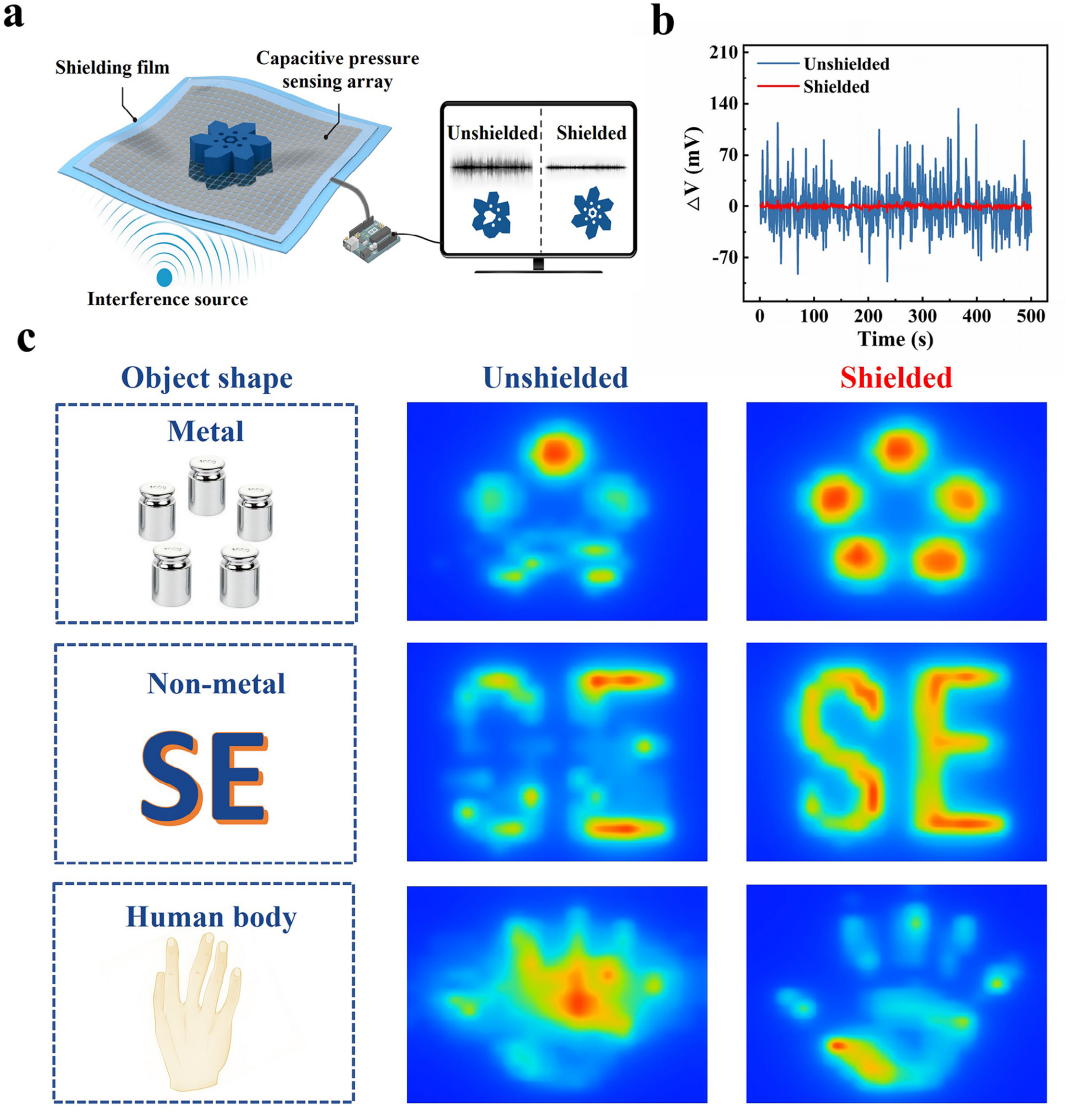
Enhanced accurate recognition of the sensor by integrating the TPU/Fe‐LM composite film into a flexible capacitive strain sensor array. a) Schematic diagram of recognizing static object by array sensor after being integrated with shielding film. b) Comparison of voltage output signals of the sensor without/with the shielding film. c) Comparison of images of different types of objects recognized by sensors without/with the shielding film.

**Figure 6:**
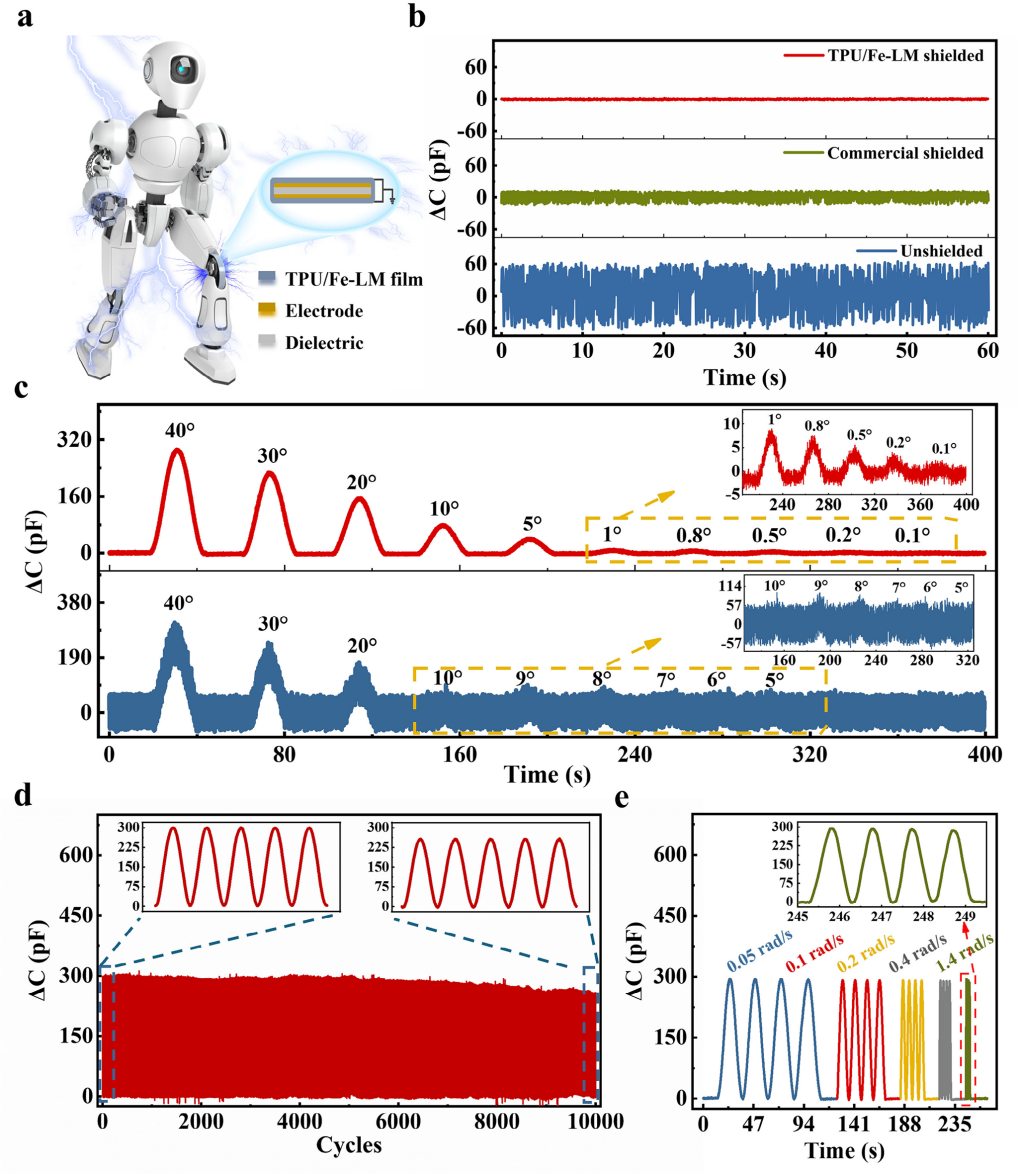
Enhanced perception capabilities of the sensor by integrating the TPU/Fe‐LM composite film into a stretchable capacitive strain sensor a) Schematic diagram of the sensor used to recognize the robot joint motions. b) Capacitance variation of shielded and unshielded sensors in static state. c) Limit detection of the robot joint angle using shielded and unshielded sensors. d) Capacitance variation of the sensor placed at the robot joint within 10000 cycles of bending motions (this bending motion was executed by the robot joint). e) Capacitance change of the shielding sensor under different motion speeds of the robot joint.

The mutual interference between sensor arrays can result in signal distortion, thus limiting the ability for accurate recognition.^[^ ^50^, ^51^, ^52^ ^]^ After integrating the TPU/Fe‐LM composite film into a flexible capacitive strain sensor array (as shown in **Figure** **5**a,b), the voltage fluctuation range of the sensor is drastically reduced from ≈±100 mV to ±7 mV. This significant reduction enhances the stability of the output voltage and effectively shields the sensor from external EMI. As shown in Figure 5c and Movies S1–S3 (Supporting Information), when metal, non‐metal, and human palms are placed on the sensor surface, the shielded sensor exhibits improved resolution. Furthermore, stretchable strain sensors endow robots with various capabilities including human‐like perception, adaptive adjustments and accurate operations.^[^ ^53^, ^54^, ^55^ ^]^ Key parameters of sensors such as precision and sensitivity directly affect the perception ability and intelligence level of robots. However, these parameters are influenced by EMI from the internal electronic components of robots and the surrounding environment, which can lead to performance degradation and system malfunction.^[^ ^56^, ^57^ ^]^ By incorporating the TPU/Fe‐LM composite film into a stretchable capacitive strain sensor placed at the robot joints (as shown in **Figure** **6**a; Movie S4, Supporting Information), the noise is reduced from ±62 pF (before shielding) to ±1.5 pF. Compared to commercially available flexible EMI shielding films, which exhibit a noise level of ±11 pF, the TPU/Fe‐LM composite film achieves nearly tenfold improvement in noise suppression (Figure 6b). The TPU/Fe‐LM composite film also demonstrates excellent durability in real‐time monitoring of the robot joint angles, achieving a resolution of ≈0.2° (Figure 6c). This represents nearly a 50‐fold improvement in resolution compared to sensors without the composite films (10° resolution). After robot joint bends by 40° for 10000 cycles, the film maintains effective performance stable ability to reduce noise (Figure 6d), demonstrating its long‐term reliability in robotic applications. Additionally, the noise reduction capability of the film was evaluated under various robot motion modes, such as walking and running. The sensor output signals remain clear and stable at bending velocities ranging from 0.05 to 1.4 rad s^−1^ (Figure 6e), further validating the reliability of the composite film for noise reduction and its potential for accurate monitoring in intelligent robot systems.

## 3 Conclusion

In summary, this study successfully developed an ultrathin stretchable EMI shielding film based on the magnetoelectric synergistic effect and the pinning‐interlocking mechanism between the ferromagnetic nanofiber network and the embedded LM. The composite film, with a thickness of only 85 µm, exhibits an average EMI SE exceeding 70 dB across a wide frequency range from 0.1 MHz to 40 GHz. Under 100% tensile strain, the EMI SE of the film changes by only 2.59%, demonstrating excellent strain stability. These outstanding performances are attributed to the 3D magnetic elastic fiber network, which ensures uniform wetting and spreading of LM, significantly enhancing the electrical conductivity of the composite film. Additionally, the magnetic loss of the ferromagnetic particles further improves the shielding performance. Furthermore, the pinning‐interlocking effect of the ferromagnetic particles effectively suppresses interface delamination between LM and magnetic fibers during tensile deformation, significantly enhancing the strain stability of the composite structure. The ultrathin composite film was integrated into a stretchable capacitive strain sensor for robot angle monitoring, improving the resolution from 10° to 0.2°, a 50‐fold increase. These advancements present a novel strategy for developing ultrathin EMI shielding materials that maintain high performance under tensile deformation. It provides a practical solution for protecting stretchable electronic devices from EMI in complex electromagnetic environments.

## 4 Experimental Section

### Materials

The high purity metal of gallium (99.99%) and indium (99.99%) were purchased from Beijing Founder Star Sci. & Technol. Co., Ltd, which were used to prepare the EGaIn eutectic alloy. Thermoplastic polyurethane elastomers (TPU) were used to prepare flexible stretchable fibers because of their great strength, toughness and solubility, which were purchased from Sigma‐Aldrich. The isopropanol alcohol (AR) and nanometer ferromagnetic particles (99.9%, 50 nm) were provided by Shanghai Macklin Biochemical Technology Co., Ltd. The solvents of N, N‐dimethylformamide (DMF, AR) and Tetrahydrofuran (THF, AR) were purchased from Shanghai Aladdin Biochemical Technology Co., Ltd.

### Preparation of EGaIn Alloy

EGaIn eutectic alloy (Ga_75_In_25_) was prepared by mixing gallium and indium in a ratio of 75 and 25 wt.%. The mixture was then heated and stirred at 85 °C for 4 h under the vacuum environment.

### Preparation of EGaIn Slurry

The EGaIn suspension was prepared by sonicating 10 g of as‐prepared bulk EGaIn eutectic alloy in 100 ml of isopropanol alcohol for 30 min, which was conducted in an ultrasonic cell disruption system at 45% amplitude (BILON 650Y, Shanghai BILON Co., Ltd.). After standing for 30 min, the LM slurry separated from the lighting suspension.

### Preparation of TPU/Fe Homogeneous Mixture

The TPU/Fe composite was prepared by dissolving TPU particles (2.16 g) in a mixed solvent of DMF/THF (12 ml) with a volume ratio of 1:1 and simultaneously mixing different mass percentages of nanometer ferromagnetic particles (10, 30, 50, and 70 wt.%). The mixture was then stirred at room temperature for 36 h to form the homogeneous TPU/Fe solution.

### Preparation of TPU/Fe‐LM Composite Films

The as‐prepared TPU/Fe solution and LM slurry were transferred to the plastic syringes with 19 G and 18 G metal nozzles, respectively. Then they were installed on both sides of the injection pump of the electrostatic spinning device (TD3030, Tianjin YunFan Technology Co., Ltd.) for electrostatic spinning and electrostatic spraying. The TPU/Fe nanofiber was produced under a positive voltage of 9 kV, and the EGaIn slurry was sprayed uniformly on the TPU/Fe nanofiber under a positive voltage of 7 kV, which were both collected on a revolving roller wrapped with aluminum foil under a negative voltage of 2 kV. The needle‐collector distances were 10 and 8 cm for TPU/Fe and EGaIn, respectively. Then pump rate for electrospinning and electrospraying was adjusted to be 0.05 and 0.02 mm s^−1^, respectively. The as‐prepared multilayered magnetic TPU/Fe fiber LM composite film was stripped from the release paper for further characterization. A positive voltage of 9 kV was applied to the needle (19 G), and a negative voltage of 2 kV was applied to the roller collector with a rotation rate of 140 r min^−1^. The precursor solution was continuously supplied to the needle with a feed rate of 0.005 mm min^−1^. The distance between the needle and the roller collector was 10 cm. The humidity and temperature inside the electrospinning equipment were kept at 40% and 35 °C.

### Characterizations

The morphology and element distribution of TPU/Fe‐LM composite films were characterized by field emission scanning electron microscopy (SEM, FEI, Quanta 250) and energy‐dispersive X‐ray spectroscopy (EDS, Bruker Silicon), respectively. The microstructures of the composite films were examined via X‐ray computed tomography (Micro‐CT, Carl Zeiss, Xradia 610). X­ray diffraction (XRD) patterns of the prepared composite films were characterized by an X‐ray diffractometer (D8 ADVANCE DAVINCI Germany) with Cu Kα radiation. The magnetic properties of the composite films with different contents of magnetic particles were characterized by vibrating sample magnetometer (VSM, Lakeshore7410).

### Measurements of Electrical Properties and Mechanical Properties

The conductivity of TPU/Fe‐LM composite films was characterized by the RTS‐9 four‐probe resistivity meter. The mechanical properties of the composite films were measured by a microforce universal mechanical testing machine (Instron 5943, USA). The surface roughness of the films was captured by AFM (Dimension Icon, Bruker). The resistance of the film was measured using a DC micro‐ohm meter (YP2512, YONGPENG). The cyclic stretching curves of the films were measured by a Cellscale instrument (Univert S2, Canada). An impedance analyzer (LCR, HIOKI, IM3570) was used to measure the capacitance data of the sensor when the humanoid robot was moving.

### Measurement of LM Mass Loading and Volume Ratio

To accurately measure the mass and volume ratio of LM in the composite films, both the blank control (TPU/Fe magnetic nanofiber film without LM) and the samples with various electrostatic spraying ratios of LM (all samples were prepared as 1cm × 1 cm squares)were weighed. The density of LM (*ρ*_LM_) was measured by the precision electronic auto balance (XSR204, Mettler Toledo), which was 6.25 g cm^−3^.

The weight of LM (*m*_LM_) and mass loading of LM (*ε*) in the as‐prepared composite films were evaluated using the following Equations (1) and (2).^[^ ^58^ ^]^

(1) $$m_{\mathrm{LM}}=m_{e}-m_{b}$$

(2) $$\varepsilon =m_{\mathrm{LM}}/m_{e}=1-m_{b}/m_{e}$$

where *m*_e_ and *m*_b_ represent the weight of the composite films (TPU/Fe‐LM) and blank control, respectively.

The volume ratio of the EGaIn eutectic alloy (*λ*) was calculated according to the following Equation (3):

(3) $$\lambda =V_{\mathrm{LM}}/V_{e}=\left(m_{\mathrm{LM}}/\rho_{\mathrm{LM}}\right)/V_{e}$$

where *V*_LM_ and *V*_e_ are the volumes of the EGaIn eutectic alloy and the as‐prepared composite films, respectively.

Here, *ε* and *λ* of composite films with various parameters were calculated using the above (Equations (1), (2), (3)).

### Electromagnetic Interference Shielding Measurement

EMI SE is a crucial indicator to evaluate the EMWs‐dampening capability of composite films. The EMI shielding properties of all static and stretchable samples within the frequency range of 8.2–12.4 GHz were characterized by a 2‐port vector network analyzer (N5234A, Keysight) with rectangular waveguides. The EMI shielding properties of the sample (Figure 2e) were characterized by a vector network analyzer (N5063A, Keysight) with a coaxial method within the frequency range of 0.1 MHz – 40 GHz. During the EMI testing, all the composite films were cut into a rectangular strip with the size of 70 × 50 mm and fixed to the self‐designed tensile stretcher, and the stretching direction was parallel to the E‐field of the EMW. The EMI SE was evaluated according to the following Equation (4):^[^ ^12^, ^32^ ^]^

(4) $$SE_{T}=SE_{R}+SE_{A}+SE_{M}$$

where SE_T_, SE_A_, SE_R_ and SE_M_ represent the total, reflection, absorption, and multiple internal reflection loss, respectively. Generally, the multiple internal reflection (SEM) can be neglected when SE_T_ is greater than 20 dB or SE_A_ is greater than 15 dB. Therefore, SE_T_ is simplified to the sum of SE_R_ and SE_A_, which can be expressed as:

(5) $$SE_{T}=SE_{R}+SE_{A}$$

In a laboratory, the SE_T_, SE_R_, and SE_A_ can be derived from the scattering parameters of *S*_11_ and *S*_21_, the scattering parameters were measured by the VNA, and calculated by the following Equations ((6), (7), (8)):

(6) $$SE_{T}=10\;\log{\left(1/|S_{21}|\right)}^{2}$$

(7) $$SE_{R}=10\;\log\left[1/\left(1-|S_{11}{|}^{2}\right)\right]$$

(8) $${\mathrm{SE}}_{A}=10\;\log\left[\left(1-|S_{11}{|}^{2}\right)/|S_{21}{|}^{2}\right]$$

### Application Demonstration on Flexible Sensors

The TPU/Fe‐LM composite film was placed between the flexible capacitor sensor array (ESPC20 × 20, ElasTech Co., Ltd.) and the interference source to monitor variations in the output voltage of the sensor. The film was also hot‐pressed at 120 °C for 20 s and incorporated into an stretchable capacitive strain sensor (RH‐ESSB‐01, ElasTech Co., Ltd.) to measure capacitance changes during the joint movements of the robot.

## Conflict of Interest

The authors declare no conflict of interest.

## Supporting information



Supporting Information

Supplemental Movie 1

Supplemental Movie 2

Supplemental Movie 3

Supplemental Movie 4

## Data Availability

The data that support the findings of this study are available in the supplementary material of this article.
