# Supplementary material for: High‐Performance, Strain‐Stable Electromagnetic Shielding Materials Enabled by Magnetic Elastic Fiber Networks Pinning Liquid Metal
Source: Adv Sci (Weinh). 2025 Jul 18;12(38):e10078. doi: 10.1002/advs.202510078 (PMC12520476; doi:10.1002/advs.202510078)
Supplement: Supplementary file 1 — Supporting Information [file ADVS-12-e10078-s004.docx]

Supporting Information

High-performance, Strain-stable Electromagnetic Shielding Materials Enabled by Magnetic Elastic Fiber Networks Pinning Liquid Metal

Qi Zhang^1,2^, Yuanzhao Wu^1^*, Xilai Bao^1^, Shengbin Li^1^, Xueheng Zhuang^1,2^, Zidong He^1^, Jinyun Liu^1^, Wuxu Zhang^1^, Shiying Li^1^, Feng Xu^1^, Chuibin Zeng^1^, Chao Hu^1^, Qikui Man^1^, Jie Shang^1^, Yiwei Liu^1^*, Run-Wei Li^3^*

^1^Ningbo Institute of Materials Technology and Engineering, Chinese Academy of Sciences, Ningbo 315201, P. R. China

^2^College of Materials Science and Opto-Electronic Technology, University of Chinese Academy of Sciences, Beijing 100049, P. R. China

^3^Eastern Institute of Technology, Ningbo, 315200, P. R. China.

Corresponding author: Yuanzhao Wu, Yiwei Liu, Run-Wei Li

E-mail: [wuyz@nimte.ac.cn](mailto:wuyz@nimte.ac.cn) (Dr. Y. Z. Wu); liuyw@nimte.ac.cn (Prof. Y. W. Liu); runweili@nimte.ac.cn (Prof. R.-W. Li)

Telephone: +86-574-87617212

Fax: +86-574-87617212


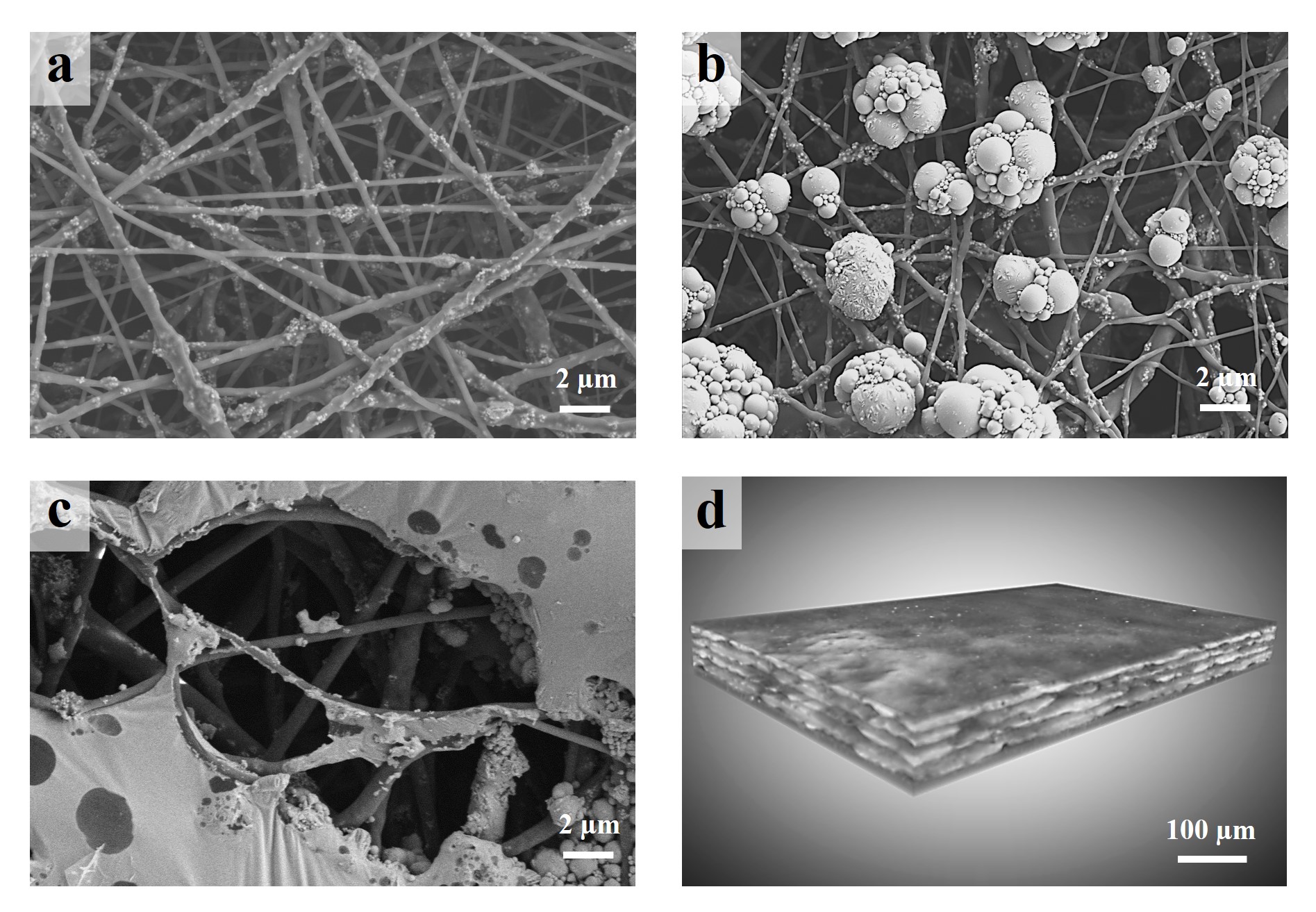


**Figure S1. Microstructures of the films**: a-c) SEM characterization of **a**) TPU/Fe film. **b**) TPU/Fe-LM composite film before mechanical activation. **c**) TPU/Fe-LM composite film after mechanical activation. **d**) 3D reconstructed morphologies of TPU/Fe-LM films by micro-CT.


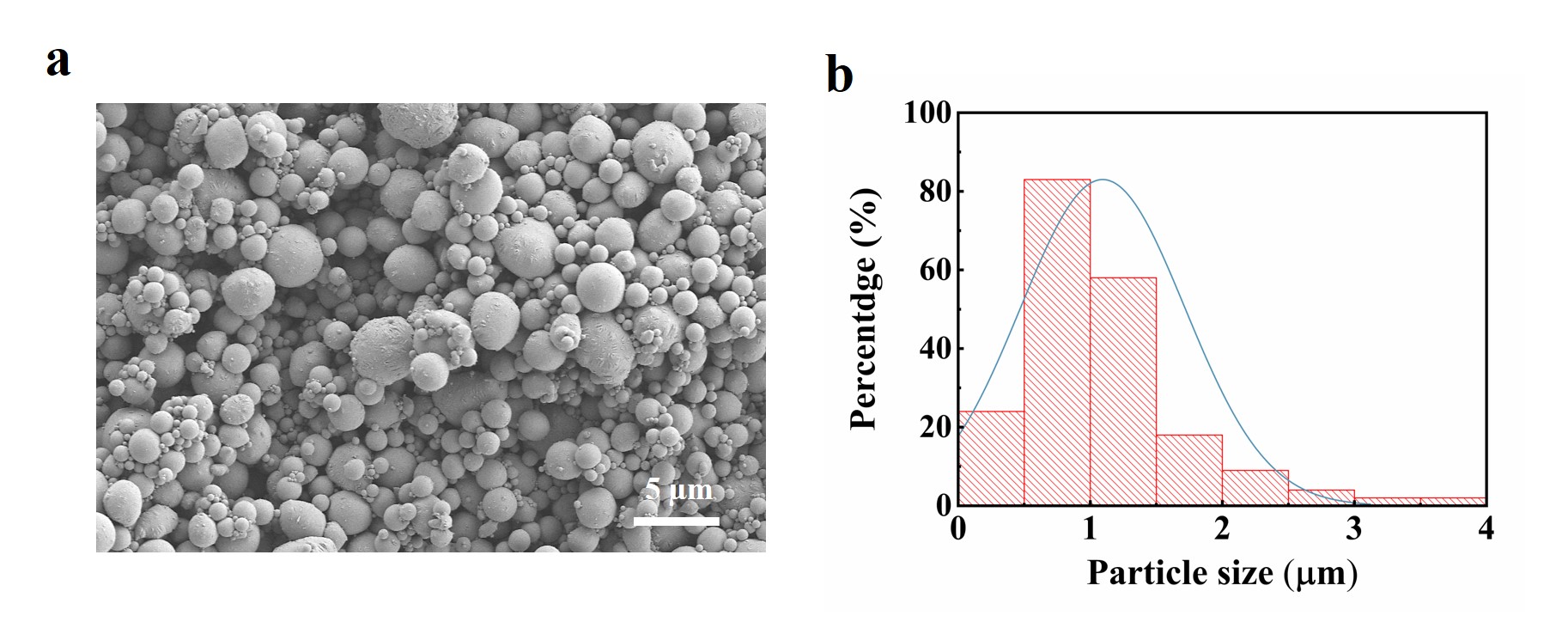


**Figure S2. Micromorphology and distribution of the LM particles**. **a)** Micromorphology. **b)** Particle size distribution.





**Figure S3.** XRD patterns of pure TPU, TPU-LM, and TPU/Fe-LM composite films.

**Table S1. Comparison of EMI shielding performances of TPU/Fe-LM composite films and other stretchable EMI shielding materials reported in the literatures**

| **Materials** | **EMI SE (dB)** | **Stretchability** | **Thickness (mm)** | **SSE (dB/mm)** | **Ref.** |  |
| --- | --- | --- | --- | --- | --- | --- |
|  |  |  |  |  |  |  |
| MXene/PU | ~21 | 0-30% | 0.26 | 80.76 | 35 |  |
| CNT/TPU | 35-13 | 0-200% | 2-0.91 | 17.5-14.28 | 36 |  |
| rGO/PU Foam | 40-7 | 0-300% | 2 | 20 | 37 |  |
| 3D LM/Ecoflex | 41.5-81.6 | 0-400% | 2-1 | 20.8-81.6 | 24 |  |
| LM/Fe/Ecoflex | 20.6-80.7 | 0-400% | 0.8-0.2 | 25-404 | 25 |  |
| LM-PDMS | 43.2-44.2 | 0-75% | 0.15-0.11 | 288.0-400.9 | 38 |  |
| AgNW/MXene/TPU/PDMS | 41.6-38 | 0-125% | ~0.3 | 138.6-126.0 | 39 |  |
| PDMS-LM Textile | 72.6-52.4 | 0-50% | 0.35 | 207.4-149.7 | 40 |  |
| LM/PDMS Lattice | ~72 | 0-100% | 9 | 8 | 34 |  |
| Cu/Microcoils/Rubber | 26.2-10 | 0-75% | 0.4 | 65.5-25.0 | 41 |  |
| PEDOT：PSS/WPU | 60-55 | 0-15% | 0.15 | 400-366 | 42 |  |
| **This work** | **~76** | **0-100%** | **0.085-0.062** | **~894-1225** | **/** |  |


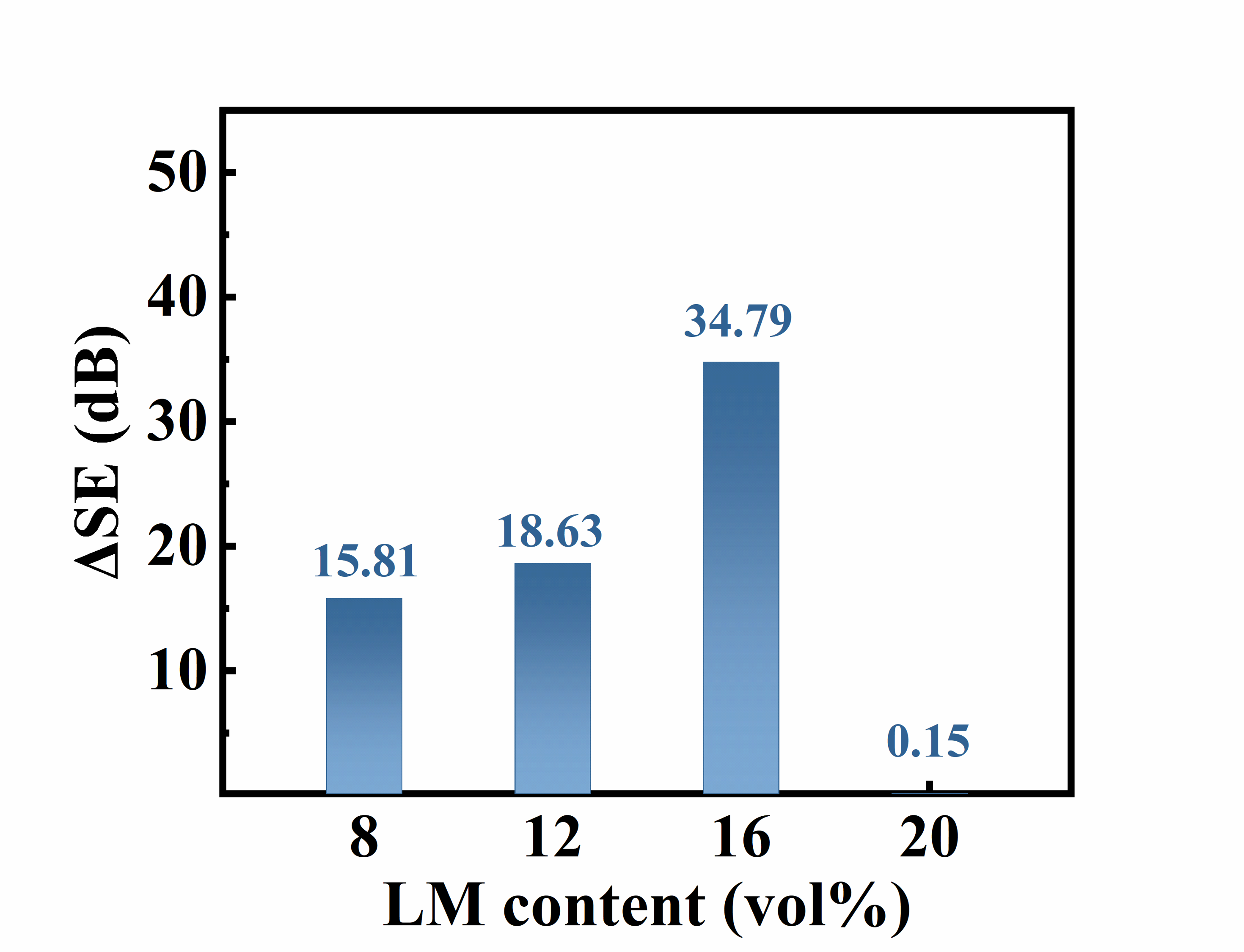


**Figure S4.** The value of increased SE after the addition of Fe particles under different LM content.





**Figure S5.** Conductivity of TPU/Fe-LM composite films with different LM contents.


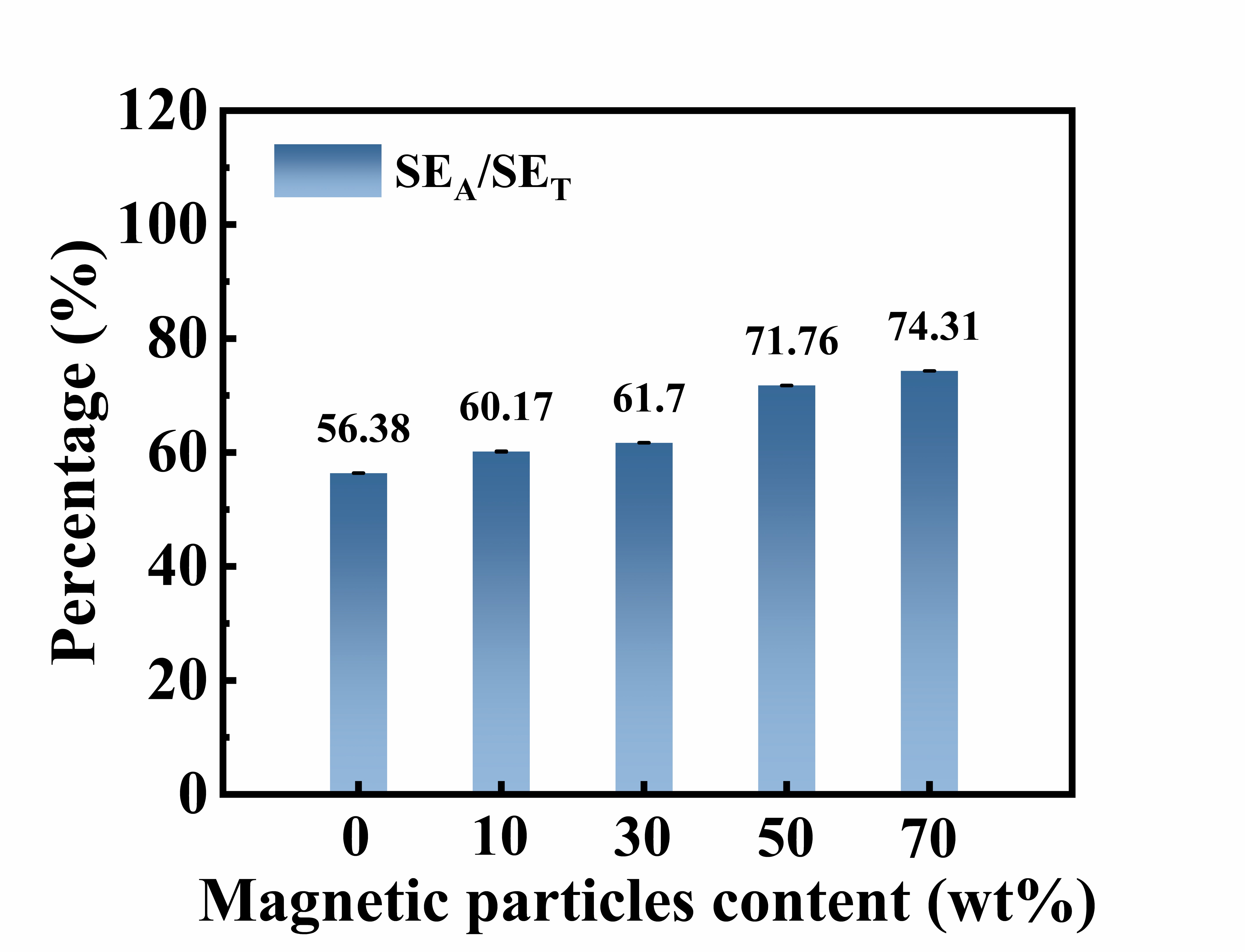


**Figure S6.** Ratio of SE_A_ to SE_T_ of TPU/Fe-LM composite films with different Fe particle contents.





**Figure S7.** Average power coefficient of reflectivity (*R*), absorptivity (*A*), and transmissivity (*T*) of TPU/Fe-LM films.


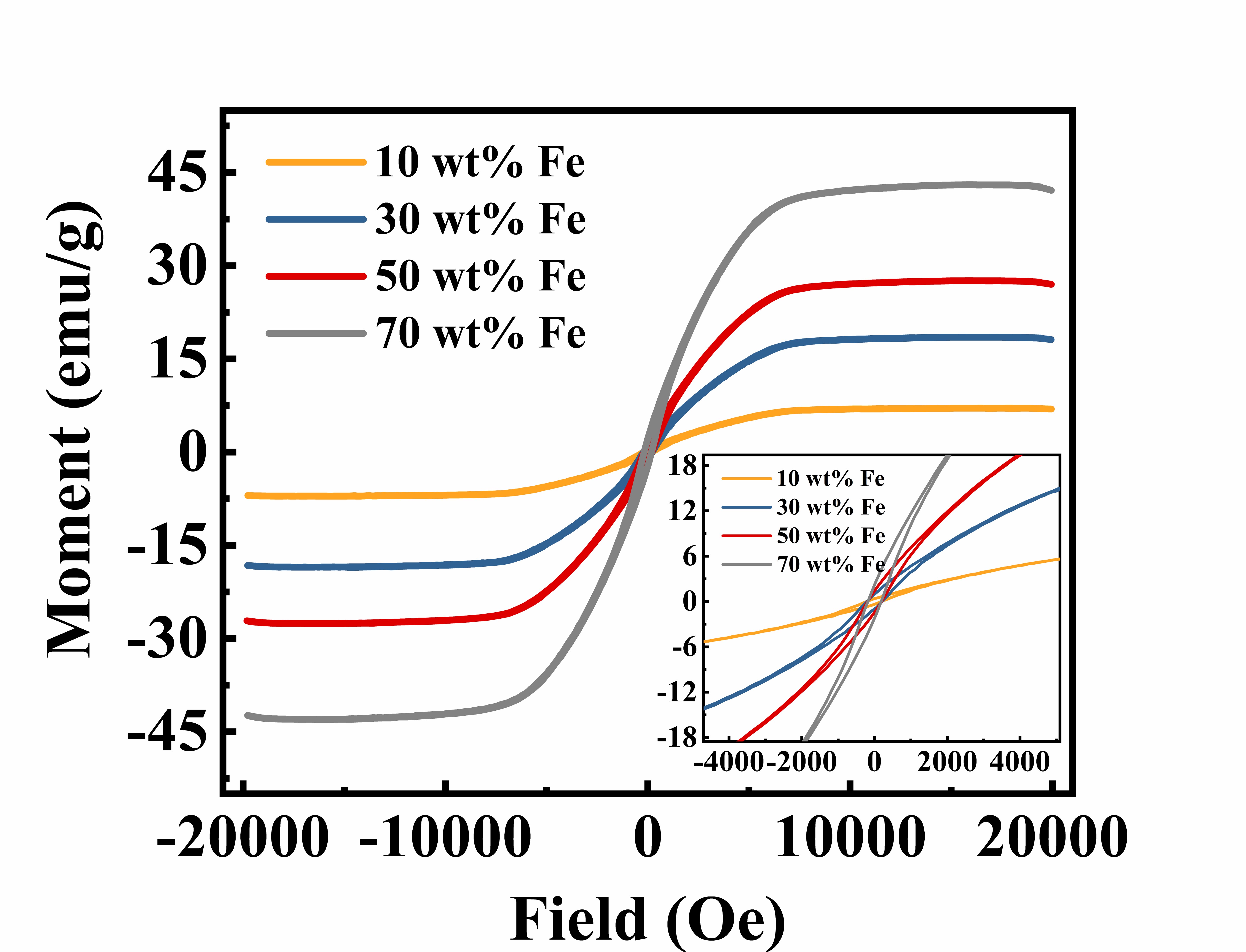


**Figure S8.** VSM curves of TPU/Fe-LM composite films with different magnetic particle contents.


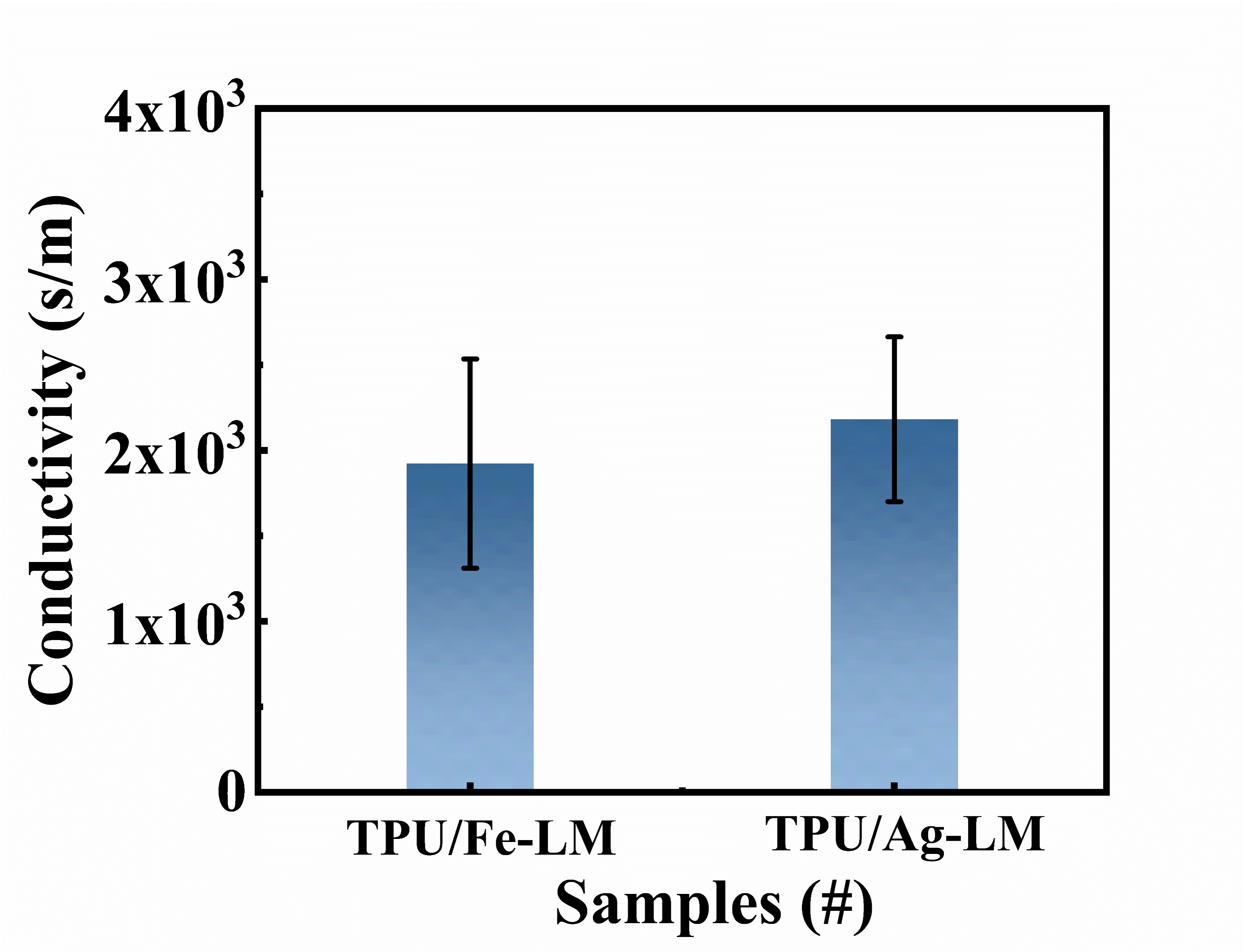


**Figure S9**. Conductivity of TPU/Fe-LM composite films and TPU/Ag-LM composite films.


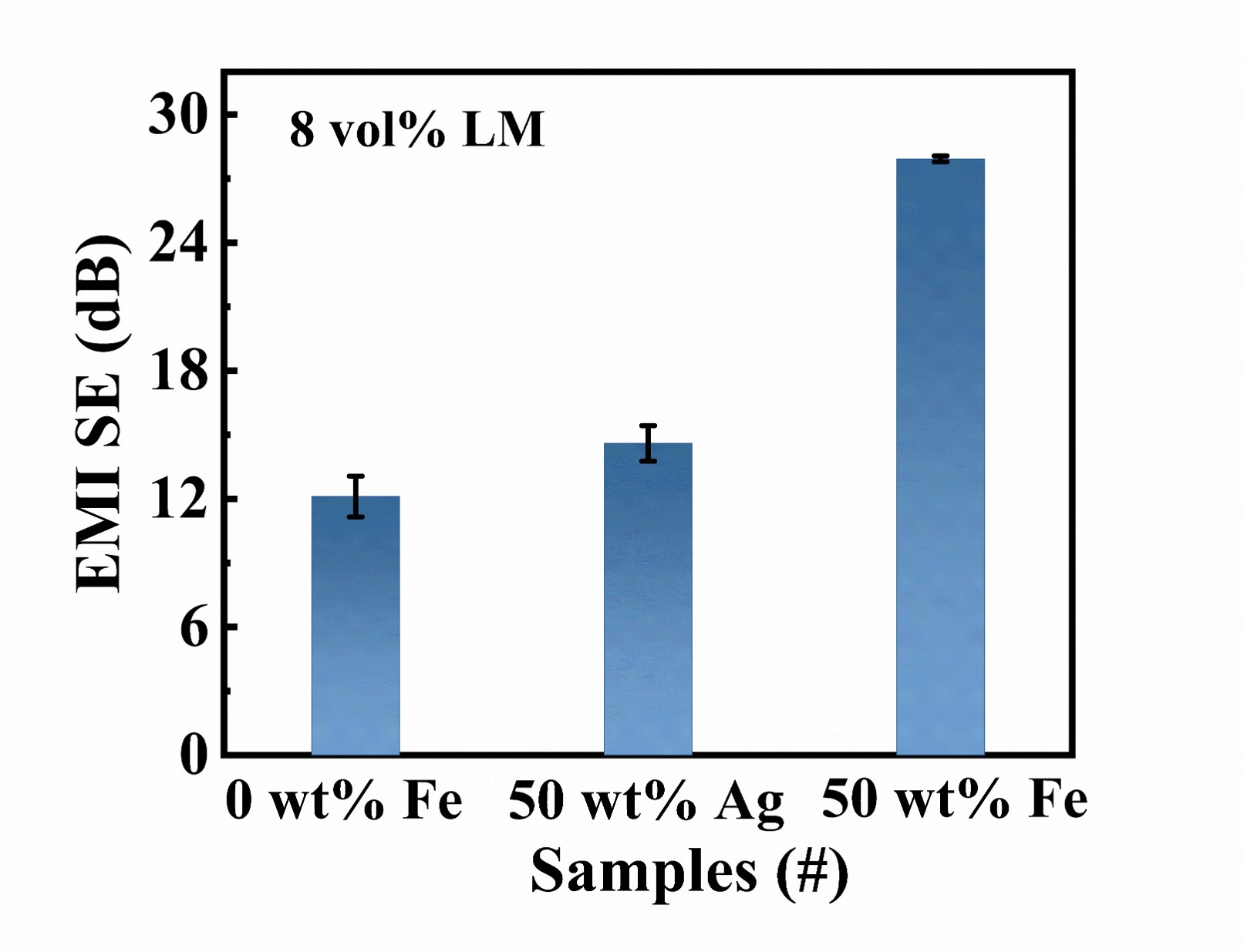


**Figure S10.** Comparison of EMI shielding performance between the films with silver powder or magnetic powder, the different films are fabricated using the same LM content.

**
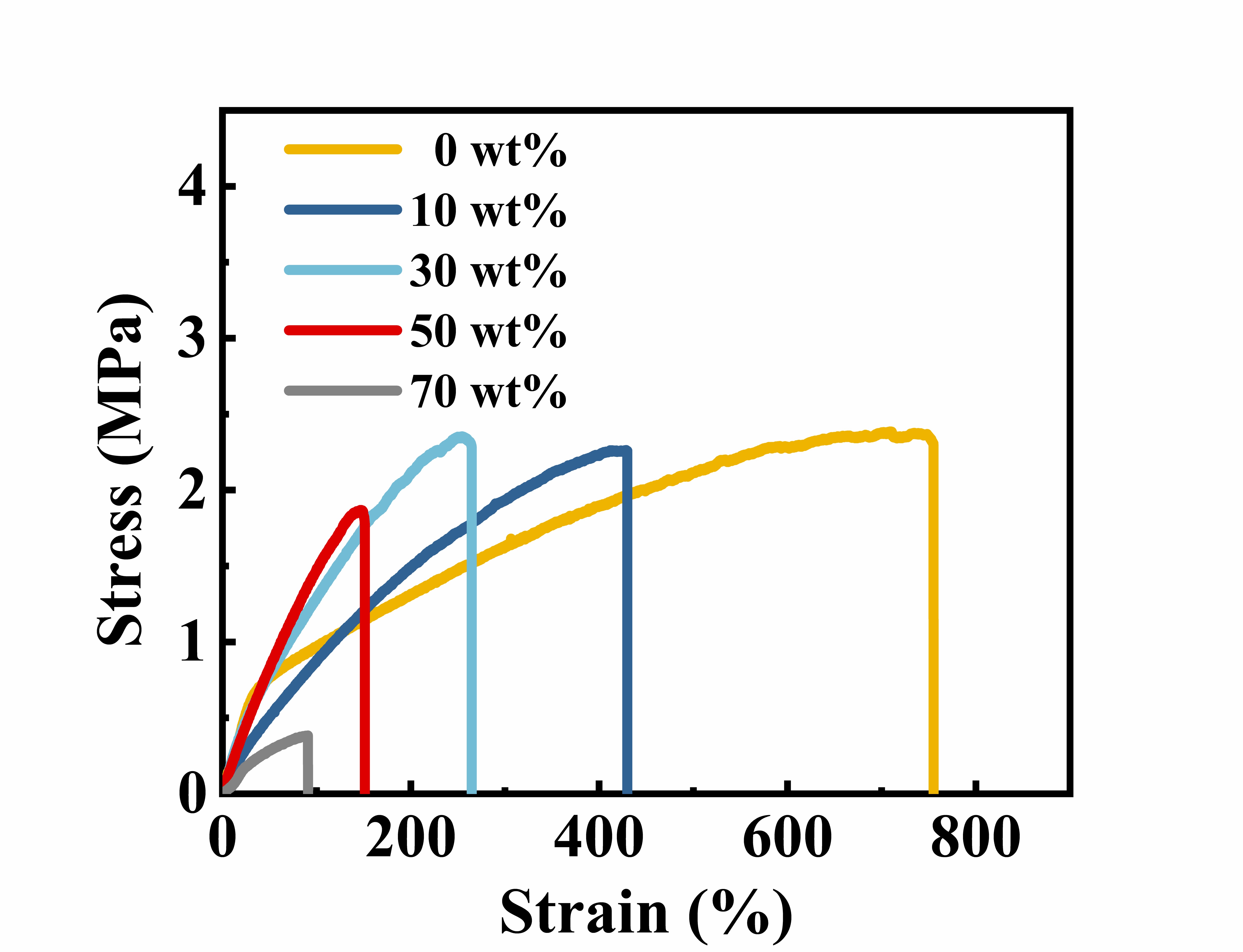
**

**Figure S11.** Stress-strain curves of TPU/Fe-LM films with different Fe particle contents.





**Figure S12**. Total EMI SE curves of TPU/Fe-LM composite films with different thicknesses over the frequency range of 8.2-12.4 GHz (the magnetic particle content of the films is fixed at 50 wt%, and the LM content of the films is fixed at 16 vol%).


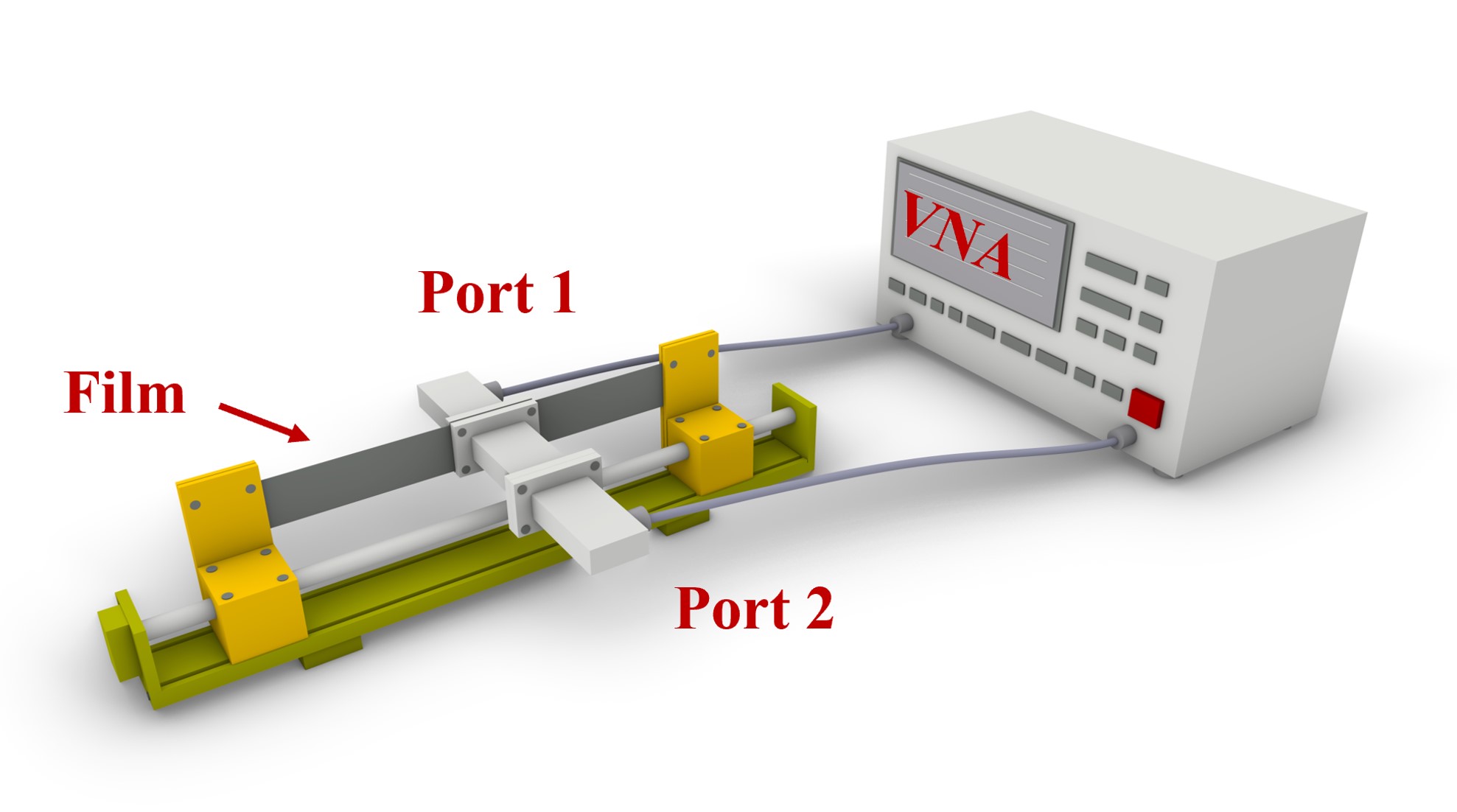


**Figure S13.** Schematic diagram of EMI shielding performance test during stretching.


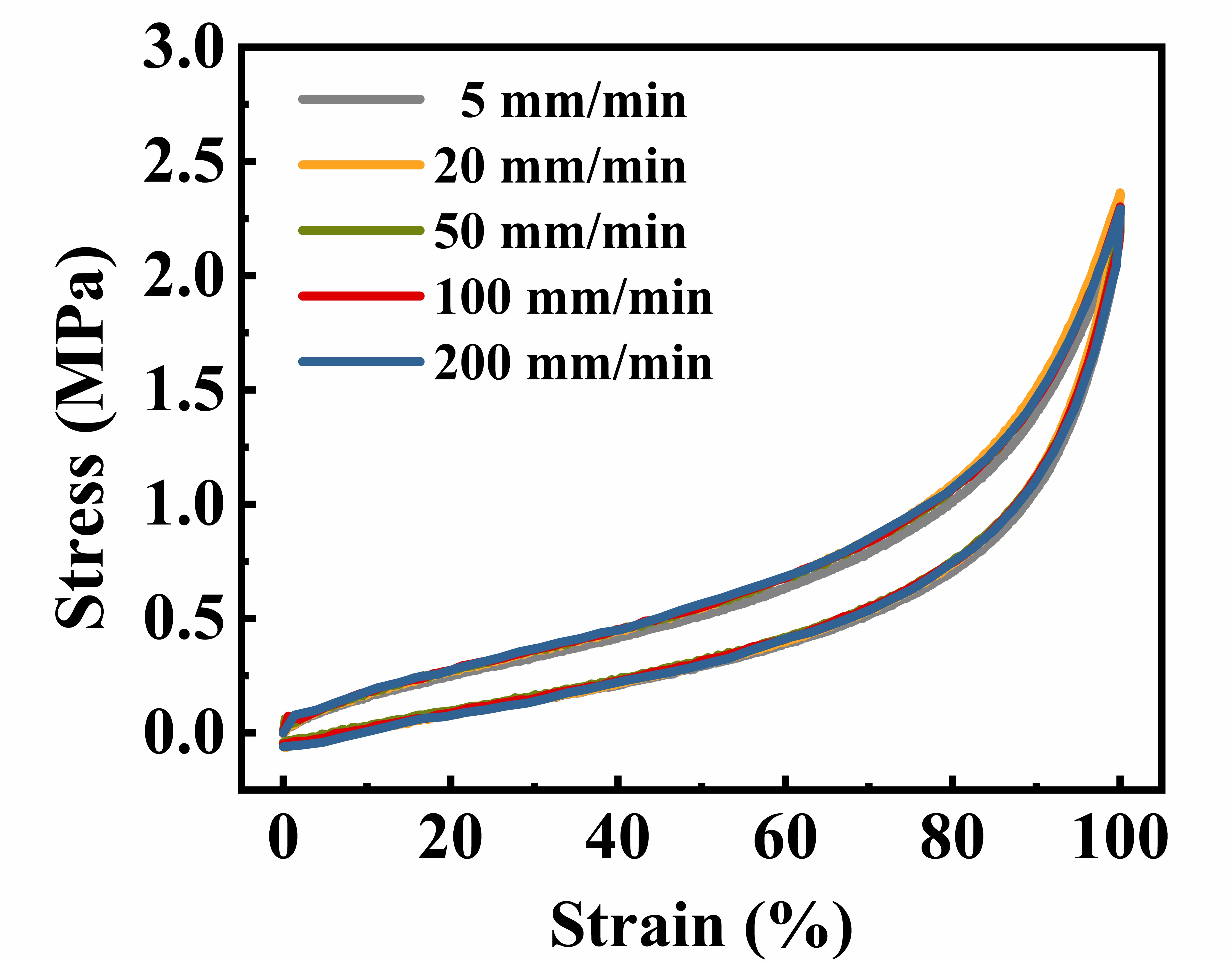


**Figure S14.** Stress-strain hysteresis loops of the TPU/Fe-LM film at various stretching speeds, the maximum strain is fixed at 100% strain (sample size: 5 mm × 10 mm).


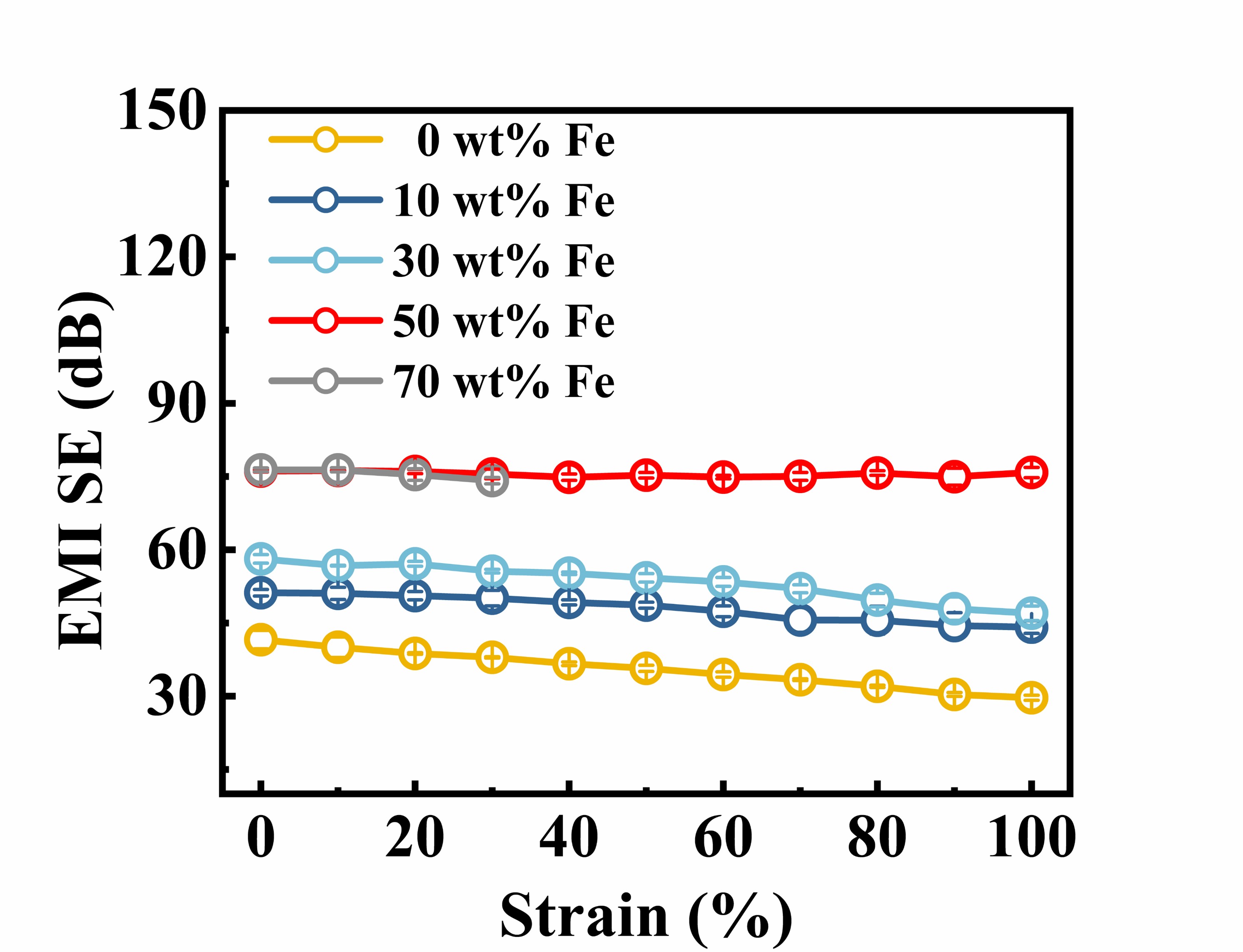


**Figure S15.** Average EMI SE versus strain of TPU/Fe-LM composite films with different magnetic particle contents, the data were averaged across the frequency ranges of 8.2-12.4 GHz.


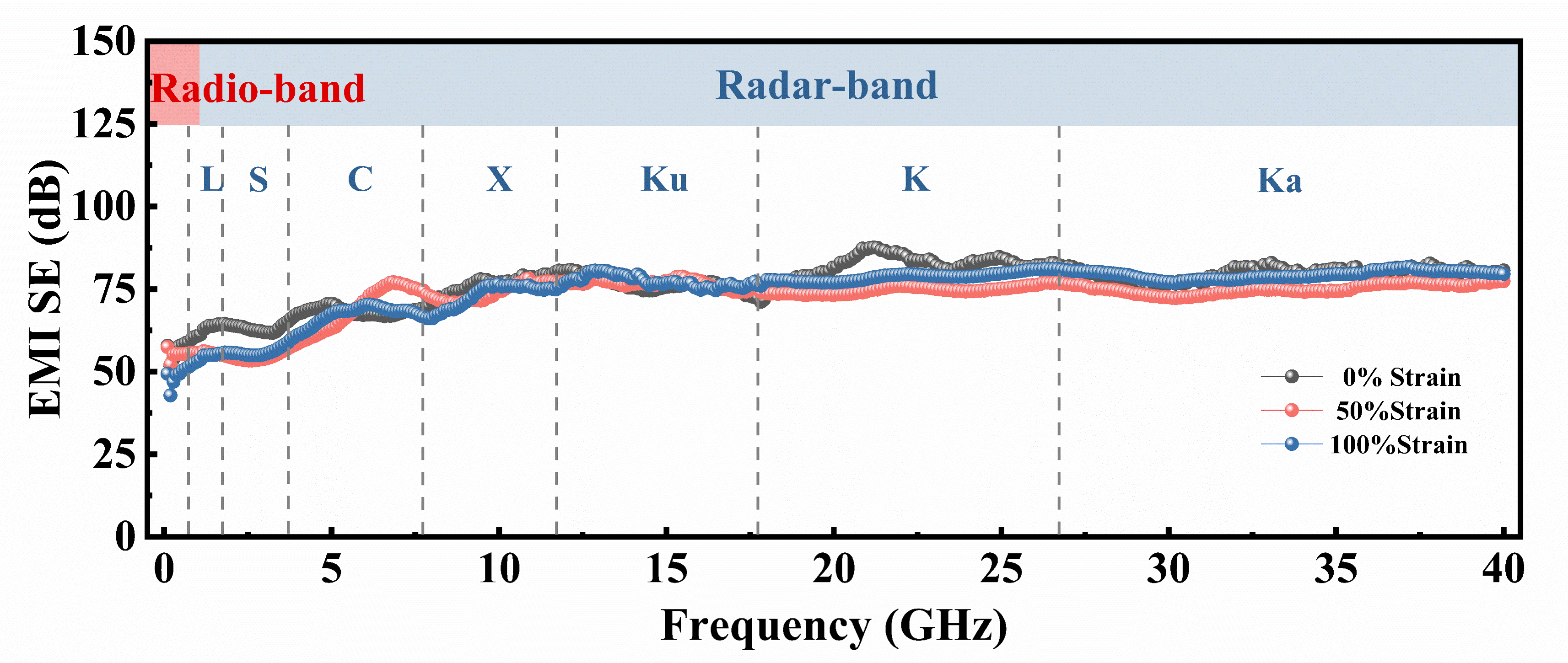


**Figure S16.** Total EMI SE curve of the TPU/Fe-LM composite film over the frequency range of 0.1 MHz - 40 GHz under 100% tensile strains.


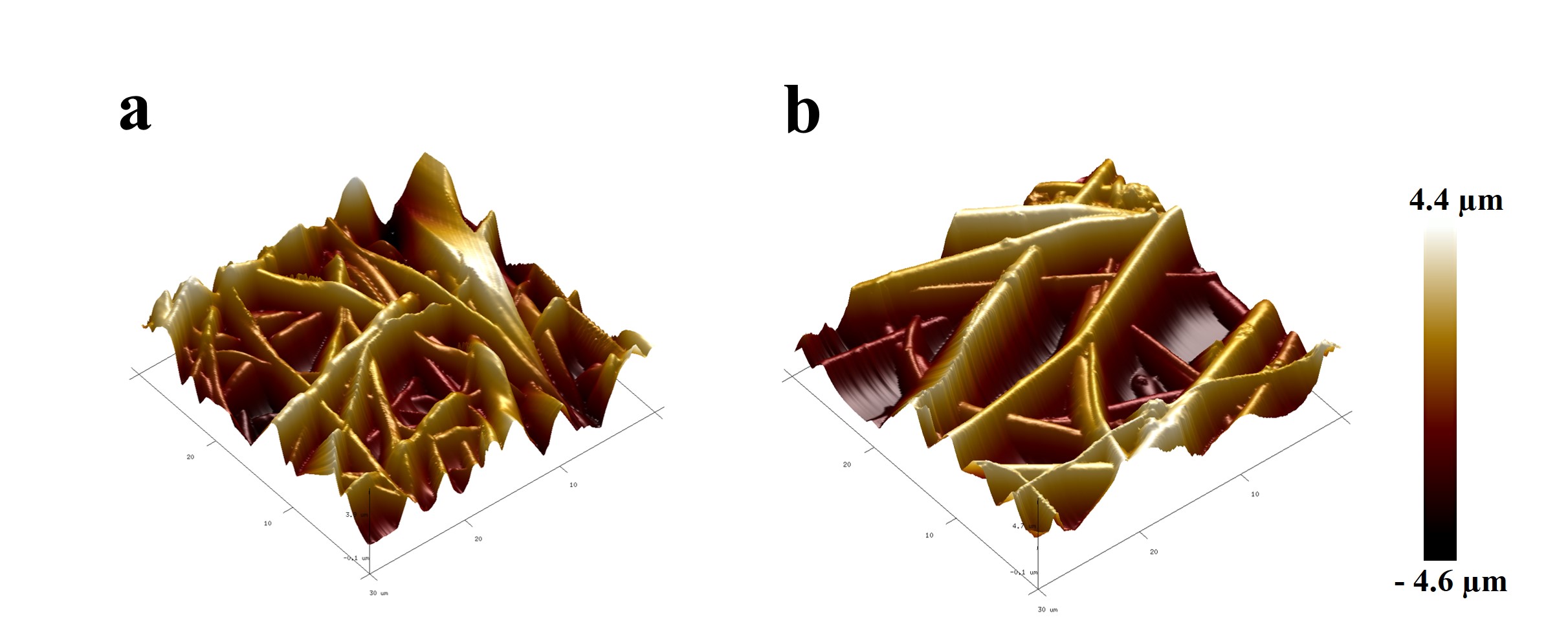


**Figure S17.** Surface roughness of TPU-LM film and TPU/Fe-LM film, the surface mappings were captured through AFM.


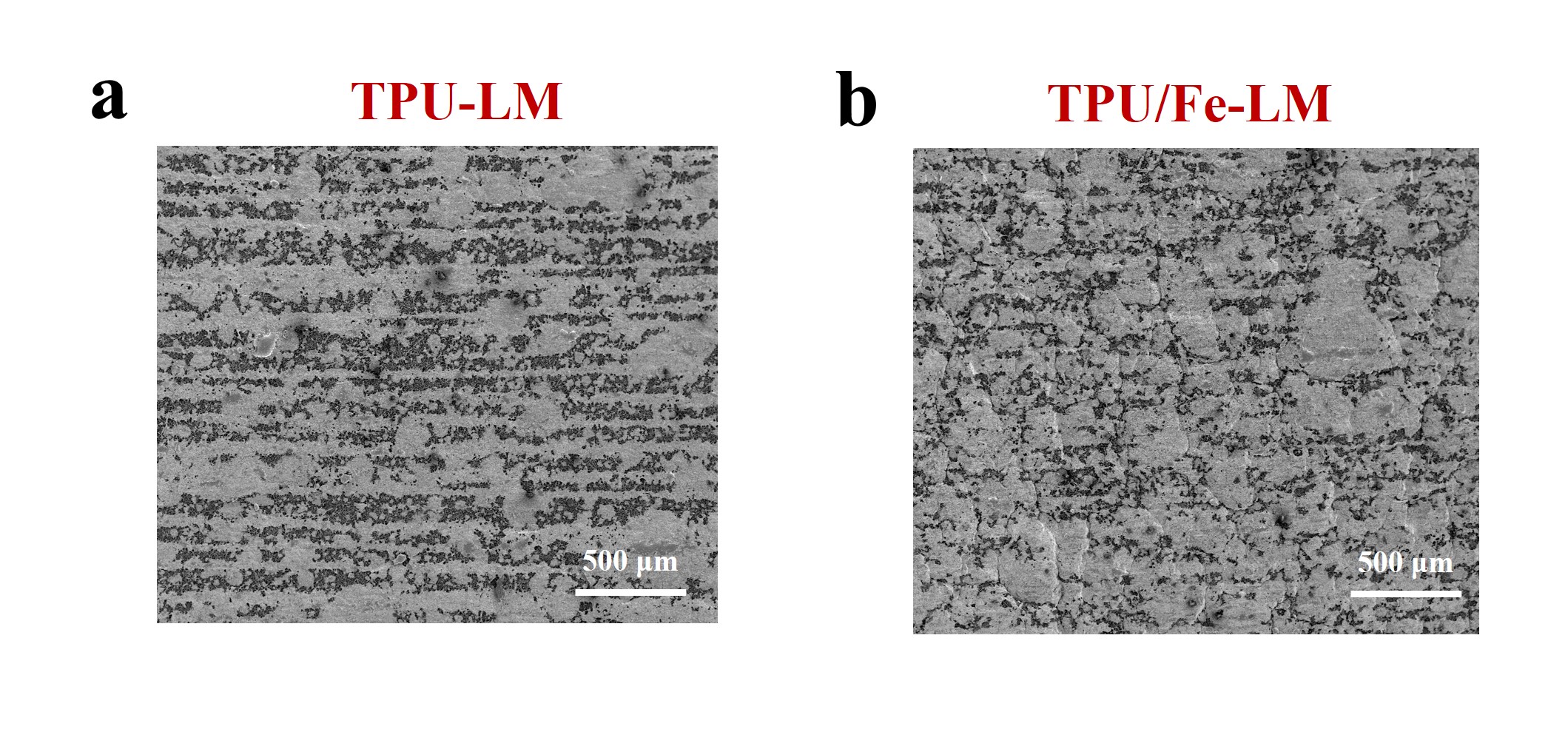


**Figure S18.** The morphology of the TPU and TPU/Fe fiber, the results show that LM spread more widely on TPU/Fe fiber.


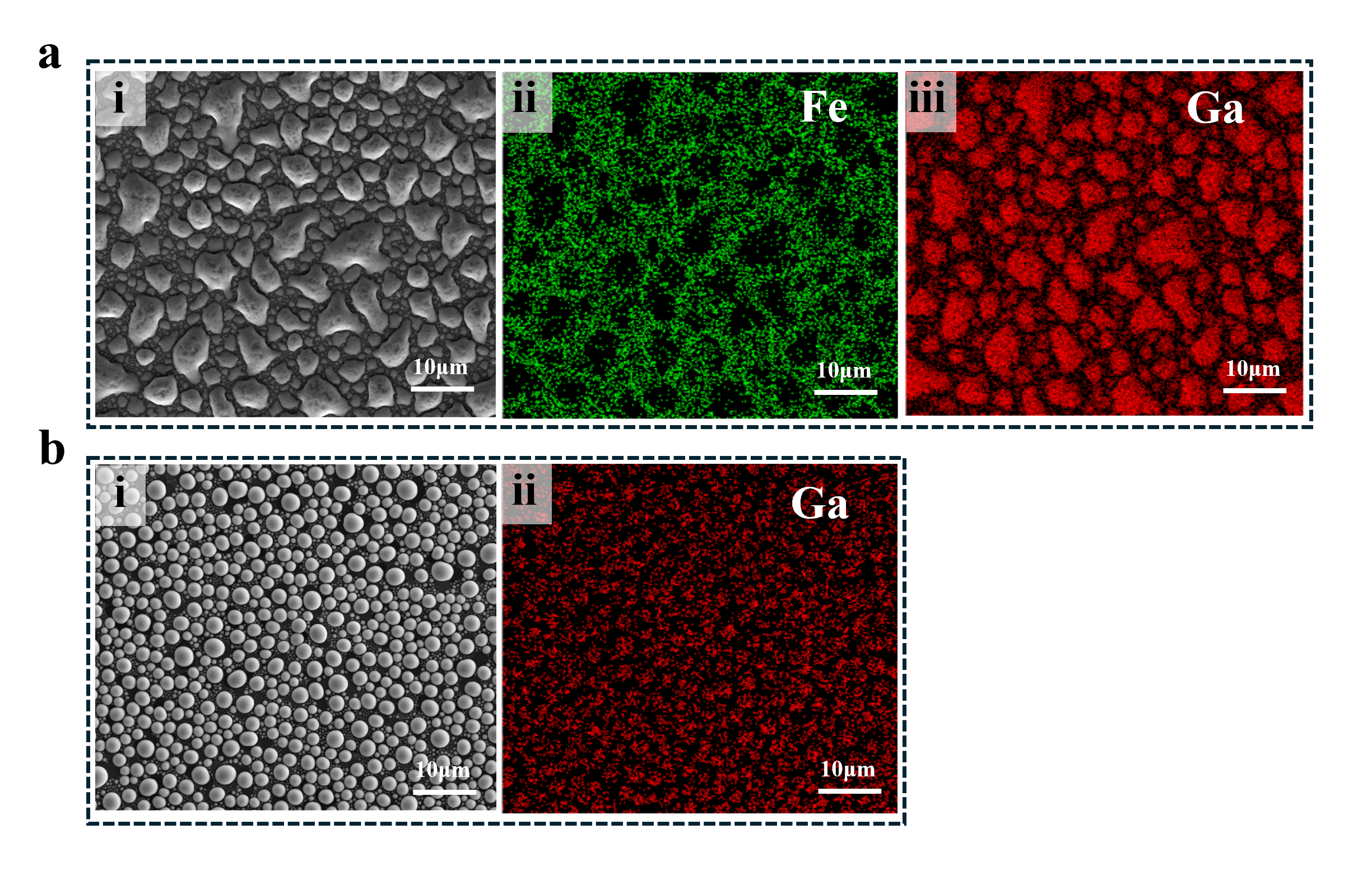


**Figure S19.** Wetting and spreading behavior of LM on iron film and polymer elastomer film. a) SEM characterization and EDS element mappings of the iron film with LM. b) SEM characterization and EDS element mappings of TPU film with LM.

To further verify the infiltration spreading behavior of liquid metal on the polymer elastomer and iron film, respectively, we designed a comparison experiment by preparing Fe nanofilms (~150 nm) on TPU substrates using electron beam deposition and LM deposition on the surface of the iron film by physical vapor deposition. The control group was LM deposition by physical vapor deposition only on the TPU surface in an amount equal to that of the experimental group. The experimental results demonstrate the significant modulation of the LM spreading behavior by the Fe film (Fig. S19). On the TPU, the LM exists in the form of isolated droplets and does not form a continuous conductive network, while on the Fe film, the LM is completely spread to form a continuous film, which indicates that the LM can be well infiltrated and spread on the Fe film.


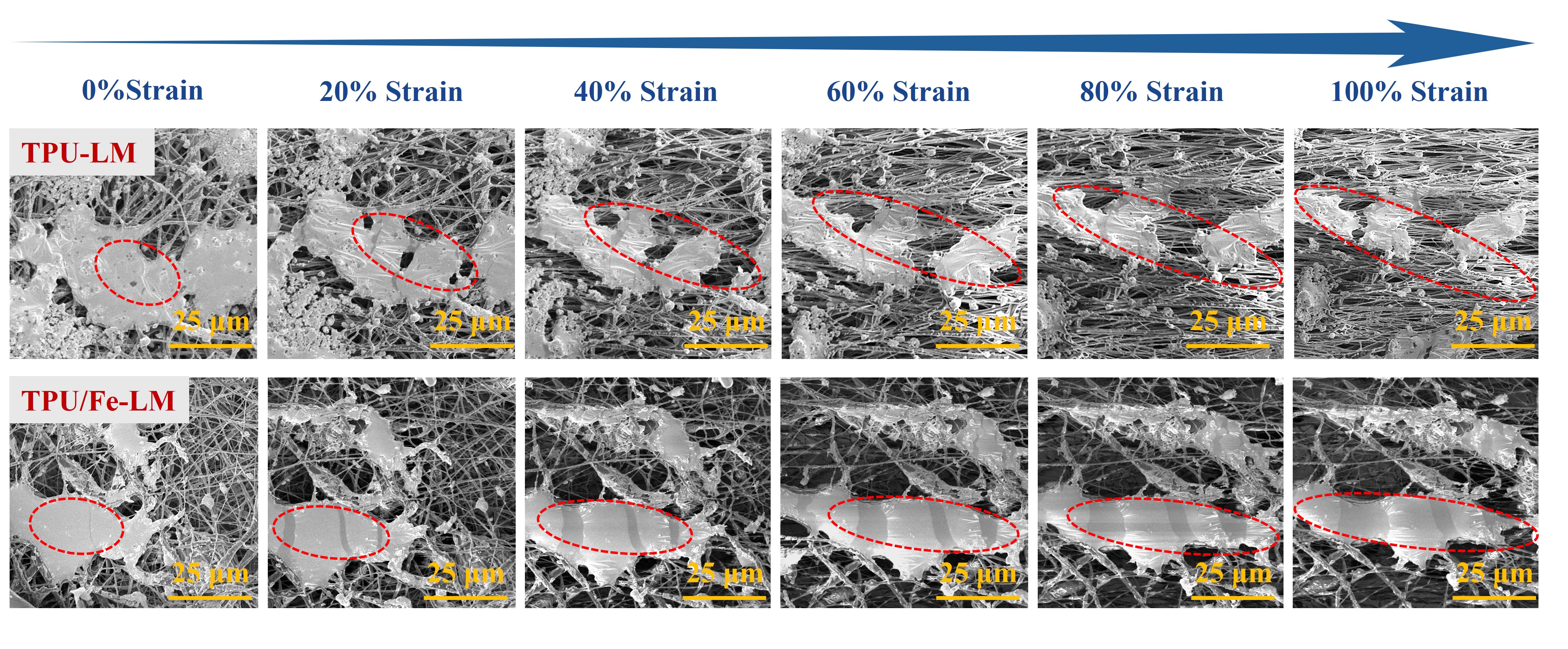


**Figure S20.** In situ micromorphology analysis of TPU-LM film and TPU/Fe-LM film under tensile deformation.

**
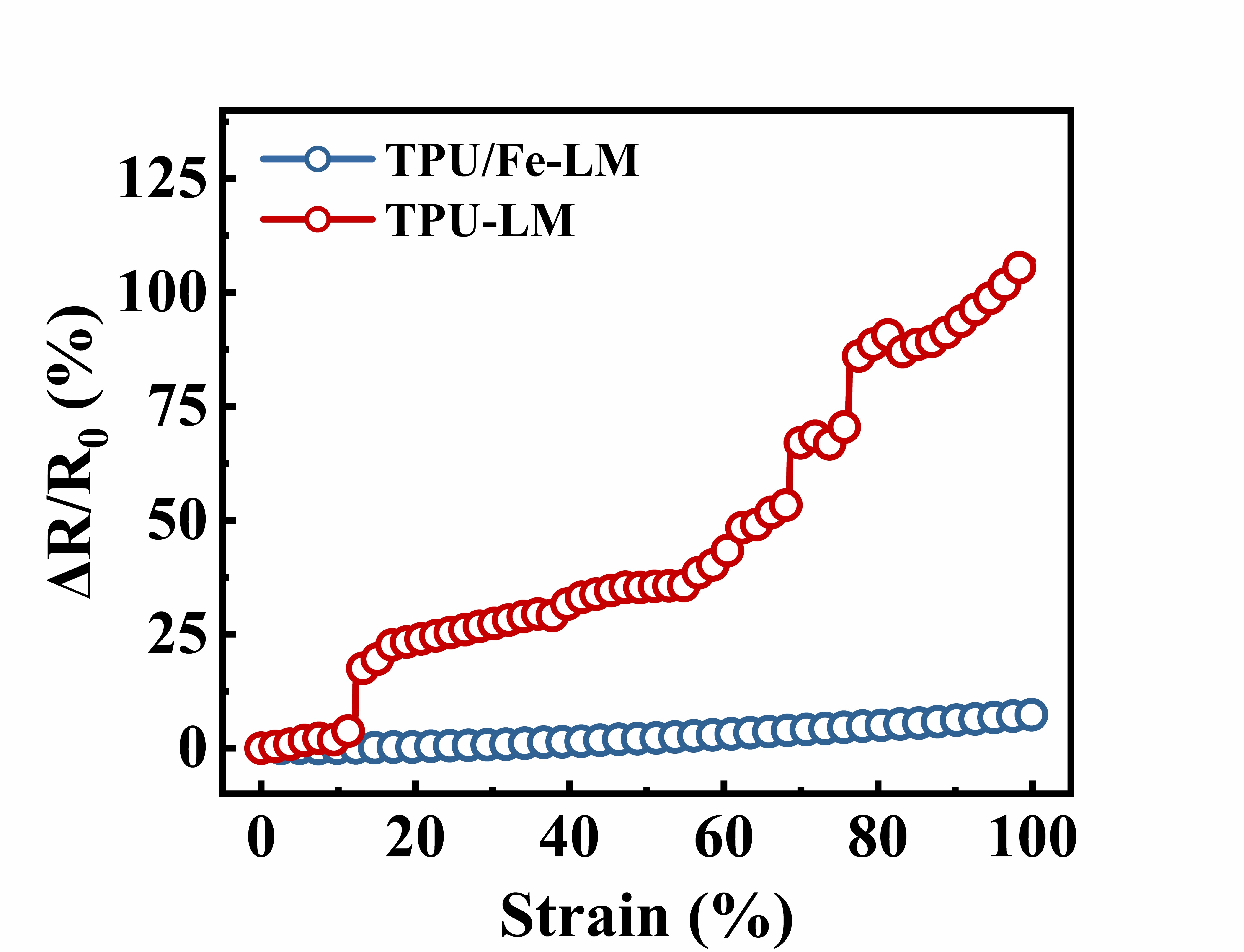
**

**Figure S21.** Comparison of resistance variation of monolayer TPU-LM film and TPU/Fe-LM film under tensile deformation.





**Figure S22.** Changes in the EMI SE of the TPU/Fe-LM film after exposure to air for one year (4 layers LM).

**Supplementary Movie:**

**Movie S1-S3.** These videos demonstrate the integration of the TPU/Fe-LM composite film onto a capacitive strain sensor array for accurate identification of metal, nonmetal, and human hands through static pressure.

**Movie S4.** This video demonstrates that integrating the TPU/Fe-LM composite film onto a capacitive elastic strain sensor enhances the resistance of the sensor to EMI and improves the perception capabilities of intelligent robotics.
